# Excess glucose alone depress young mesenchymal stromal/stem cell osteogenesis and mitochondria activity within hours/days via NAD^+^/SIRT1 axis

**DOI:** 10.1186/s12929-024-01039-0

**Published:** 2024-05-13

**Authors:** B. Linju Yen, Li-Tzu Wang, Hsiu-Huang Wang, Chin-Pao Hung, Pei-Ju Hsu, Chia-Chi Chang, Chien-Yu Liao, Huey-Kang Sytwu, Men-Luh Yen

**Affiliations:** 1 Regenerative Medicine Research Group, Institute of Cellular & System Medicine, National Health Research Institutes (NHRI), No.35, Keyan Road, Zhunan, 35053 Taiwan; 2 Department of Obstetrics & Gynecology, National Taiwan University (NTU) Hospital & College of Medicine, NTU, No.1, Section 1, Jen-Ai Road, Taipei, 10051 Taiwan; 3 School of Medical Laboratory Science and Biotechnology, College of Medical Science and Technology, Taipei Medical University, No. 250, Wuxing Street, Taipei, 11042 Taiwan; 4 Ph.D. Program in Medical Biotechnology, College of Medical Science and Technology, Taipei Medical University, No.250, Wuxing Street, Taipei, 11042 Taiwan; 5 Graduate Institute of Life Sciences, National Defense Medical Center (NDMC), No.161, Section 6, Minquan East Road, Taipei, 11490 Taiwan; 6 National Institute of Infectious Diseases & Vaccinology, NHRI, No.35, Keyan Road, Zhunan, 35053 Taiwan; 7 Graduate Institute of Microbiology & Immunology, NDMC, No.161, Section 6, Minquan East Road, Taipei, 11490 Taiwan

**Keywords:** Human mesenchymal stromal/stem cells (MSCs), Osteogenesis, Adipogenesis, Mitochondria, High glucose, Mouse model, Nicotinamide adenine dinucleotide (NAD^+^), Nicotinamide mononucleotide (NMN), Sirtuin 1 (SIRT1)

## Abstract

**Background:**

The impact of global overconsumption of simple sugars on bone health, which peaks in adolescence/early adulthood and correlates with osteoporosis (OP) and fracture risk decades, is unclear. Mesenchymal stromal/stem cells (MSCs) are the progenitors of osteoblasts/bone-forming cells, and known to decrease their osteogenic differentiation capacity with age. Alarmingly, while there is correlative evidence that adolescents consuming greatest amounts of simple sugars have the lowest bone mass, there is no mechanistic understanding on the causality of this correlation.

**Methods:**

Bioinformatics analyses for energetics pathways involved during MSC differentiation using human cell information was performed. In vitro dissection of normal versus high glucose (HG) conditions on osteo-/adipo-lineage commitment and mitochondrial function was assessed using multi-sources of non-senescent human and murine MSCs; for in vivo validation, young mice was fed normal or HG-added water with subsequent analyses of bone marrow CD45^−^ MSCs.

**Results:**

Bioinformatics analyses revealed mitochondrial and glucose-related metabolic pathways as integral to MSC osteo-/adipo-lineage commitment. Functionally, in vitro HG alone without differentiation induction decreased both MSC mitochondrial activity and osteogenesis while enhancing adipogenesis by 8 h’ time due to depletion of nicotinamide adenine dinucleotide (NAD^+^), a vital mitochondrial co-enzyme and co-factor to Sirtuin (SIRT) 1, a longevity gene also involved in osteogenesis. In vivo, HG intake in young mice depleted MSC NAD^+^, with oral NAD^+^ precursor supplementation rapidly reversing both mitochondrial decline and osteo-/adipo-commitment in a SIRT1-dependent fashion within 1 ~ 5 days.

**Conclusions:**

We found a surprisingly rapid impact of excessive glucose, a single dietary factor, on MSC SIRT1 function and osteogenesis in youthful settings, and the crucial role of NAD^+^—a single molecule—on both MSC mitochondrial function and lineage commitment. These findings have strong implications on future global OP and disability risks in light of current worldwide overconsumption of simple sugars.

**Supplementary Information:**

The online version contains supplementary material available at 10.1186/s12929-024-01039-0.

## Introduction

Excessive simple sugar/monosaccharide consumption is a global concern by strongly increasing numerous disease risks [1–7] but surprisingly its association with bone health and osteoporosis (OP), an age-related disease of bone fragility with outsized contributions to global disease disability by increasing fracture risks [8–10], has remained largely unclear. The cell responsible for bone formation is the osteoblast, a descendant cell type of the multilineage mesenchymal stromal/stem cell (MSC)—first identified in the non-hematopoietic/CD45^−^ compartment of the bone marrow—which can also differentiate into other skeletal-related lineages particularly adipocytes and chondrocytes [11, 12]. MSC osteogenesis appears to be fragile, with senescence decreasing its osteogenic capacity but increasing adipogenic capacity [13–18]. The rapid aging of populations worldwide has made OP a critical public health concern, and despite the availability of numerous treatments and recent discoveries of potent biologics, patient compliance remains problematic due to slow improvement coupled with rare but severe adverse effects in all classes of therapeutics [19]. Unlike most tissues/organs, over 90% of peak bone mass is acquired by the age of 20, decades before the onset of OP and fracture risk, with bone loss beginning after the age of 30 [20–22]. Moreover during this time period, suboptimal lifestyle choices—one of few modifiable OP risk factors—including poor dietary habits strongly increase OP and fracture risks later in life [23]. Alarmingly, there are worrisome trends of plateauing height growth and low bone mass in ever younger people, with correlative evidence showing adolescents consuming the greatest amounts of simple sugars having the lowest bone mass [24–26]. Despite global sugar overconsumption and the widening epidemic of OP, molecular understanding of how excessive simple sugar/monosaccharide intake affects MSC osteogenic lineage commitment in youthful, non-senescent settings remains largely unexplored, with the existing few mechanistic studies yielding inconsistent results [27–30].

Found in all living cells, a vital co-enzyme involved in mitochondrial function is nicotinamide adenine dinucleotide (NAD^+^) [31]. Intake of excessive or high glucose (HG), the most common monosaccharide, triggers glycolysis which depletes NAD^+^ levels by requiring NAD^+^ to accept electrons and form NADH. Under normal glucose conditions, however, mitochondrial function is maintained which improves NAD^+^ levels and also the function of Sirtuin (SIRT) 1 [32], an NAD^+^-dependent histone deacetylase with crucial roles in organismal longevity [33, 34] and MSC osteogenesis [35, 36]. While it is well known that aging and senescence decreases NAD^+^ levels and bone mass [37, 38], the more relevant questions for bone health which anabolically peaks in adolescence/early adulthood, has surprisingly not been answered: whether modulating NAD^+^ levels in young/non-senescent settings would impact MSC osteogenesis, and the role of simple sugar overconsumption on osteogenic commitment in young/non-senescent MSCs. We therefore investigated the role and any underlying mechanism of HG intake in modulating MSC mitochondrial function and lineage commitment in in vitro human and murine MSCs and in vivo. Our findings demonstrate surprisingly that HG intake alone can rapidly—within hours in vitro and 1–5 days in vivo—deplete NAD^+^ levels, decreasing mitochondrial function and switching MSC lineage commitment from osteogenesis to adipogenesis by decreasing downstream SIRT1 actions. The findings not only demonstrate the detrimental and unexpected rapid impact of HG intake alone simultaneously on MSC mitochondrial function and osteogenic commitment in non-senescent settings, but also show the implications of simple sugar overconsumption on bone health even in youthful settings.

## Methods

### Cell culture and differentiation

Human bone marrow (BM) MSCs were obtained from commercial sources (Promocell, Heidelberg, Germany), while mouse BMMSCs were isolated from C57BL/6 J strain (National Laboratory Animal Center, Taipei, Taiwan). Human placental MSCs (PMSCs) were isolated as previously reported from term placental tissue (38–40 weeks’ gestation) obtained from healthy donor mothers after informed consent approval [39]. All human MSCs were cultured in low-glucose Dulbecco’s Modified Eagle’s medium (DMEM) (Invitrogen -Thermo Fisher Scientific) with 10% FBS (Hyclone-Thermo Fisher Scientific), 100U/ml of penicillin/streptomycin, 2 mM L-glutamine (all from Gibco-Thermo Fisher Scientific) [40]. Non-senescent MSCs, including passages 8–10 for BMMSCs and passages 15–20 for PMSCs, were used as previously reported [15, 41, 42]. Mouse C3H10T1/2 MSCs were obtained from American Type Culture Collection (ATCC, Manassas, VA, USA) and maintained in Basal Medium Eagle medium (BMEM) (Invitrogen-Thermo Fisher) with 10% FBS, 100 U/ml of penicillin/streptomycin, 2 mM L-glutamine [43]. Adipogenic and osteogenic differentiation with quantification of Oil Red O and Alizarin Red staining, respectively, were performed as previously reported [15, 39].

### Bioinformatic analyses

Transcriptomic profiling databases were obtained from the National Center for Biotechnology Information-Gene Expression Omnibus database for bioinformatic analyses: GSE20631 for human BMMSCs, GSE45169 for human adipocytes, GSE101140 for human osteoblasts, and GSE128423 for single-cell RNA sequencing of uncultured BM CD45^−^ osteolineage cells and MSCs harvested from male C57BL/6 mice at age 6–8 weeks [44]. Principal component analysis (PCA) of transcriptomic profiles was performed using Partek® Flow® software (Partek, Inc., St. Louis, MO), while enriched pathways in Gene Ontology (GO) Biological Processes were performed using Gene set enrichment analysis (GSEA) which showed positive enrichment with a normalized enrichment score (NES) > 1 and a *p*-value < 0.05, as well as Metascape analysis in which significance was set at *p*-value < 0.01 [45]. The Molecular Activation Prediction (MAP) tool was used to interrogate networks and pathways with expression levels of involved genes using Ingenuity Systems Pathway Analysis (IPA) software (QIAGEN, Hilden, Germany) [46]. IPA showed regulatory relationships between up-regulatory (red) and down-regulatory (green) molecules, while the MAP tool predicted that the targeted processes or involved molecules were activated (orange) or inhibited (blue) through relationships with positive regulation (orange lines), negative regulation (blue lines), inconsistent findings (yellow lines), or being not predicted (gray lines). Dashed lines represented indirect interactions based on published data.

### Real-time PCR

RNA was extracted with TRIzol reagent (Invitrogen-Thermo Fisher Scientific) and cDNA synthesis was performed with ReverTra Ace set (TOYOBO, Osaka, JP), both according to the manufacturer’s protocols. SYBR was applied for the assessment of PCR products by ABI 7500 Fast Real-Time PCR system (Thermo Fisher Scientific). Primers for human and murine genes are listed in Table 1.

**Table 1:** The list of primer sequence for qPCR

Gene		Forward primer	Reverse primer
Human	*GAPDH* (internal control)	5'-GTGGACCTGACCTGCCGTCT-3'	5'-GGAGGAGTGGGTGTCGCTGT-3'
	*GLUT1*	5'-CATCAACCGCAACGAGGAGAAC-3'	5'-CAGCACCACAGCGATGAGGAT-3'
	*GLUT2*	5'-GCTGCCGCTGAGAAGATTAGAC-3'	5'-CCTGACTAGCTCCTGCCTGTT-3'
	*GLUT3*	5'-ACGGCCTCATGGAGAATGAACAG-3'	5'-AGCAGCATTCAGAAGCGTCCT-3'
	*GLUT4*	5'-ATCCTTGGACGATTCCTCATTGG-3'	5'-CAGGTGAGTGGGAGCAATCT-3'
	*ADIPOQ*	5'-GCTCAGCATTCAGTGTGGGA-3'	5'-GTACAGCCCAGGAATGTTGC-3'
	*IBSP*	5'-GGCCACGATATTATCTTTACAAGCA-3'	5'-CCTCAGAGTCTTCATCTTCA-3'
	*CEBPB*	5'-AAACTCTCTGCTTCTCCCTCTG-3'	5'-GTTGCGTCAGTCCCGTGTA-3'
	*KLF5*	5'-CCTGGTCCAGACAAGATGTGA-3'	5'-GAACTGGTCTACGACTGAGGC-3'
	*RUNX2*	5'-CCAGATGGGACTGTGGTTACTG-3'	5'-TTCCGGAGCTCAGCAGAATAA-3'
	*ALPL*	5'-AGCTGAACAGGAACAACGTGA-3'	5'-CTTCATGGTGCCCGTGGTC-3'
Murine	*Actb* (internal control)	5'-GAAATCGTGCGTGACATCAAAG-3'	5'-TGTAGTTTCATGGATGCCACAG-3'
	*Glut1*	5'-GGTGCCAGCCAAAGTGACAAC-3'	5'-GTCGGTTCGGAAGAGGTCTCAT-3'
	*Glut2*	5'-TTCCAGTTCGGCTATGACATCG-3'	5'-CTGGTGTGACTGTAAGTGGGG-3'
	*Glut3*	5'-CGGTGTGGAGTTGAACAGCAT-3'	5'-ATAGCAGCACTCAGAAGCAGTC-3'
	*Glut4*	5'-AGCCAGCCTACGCCACCATA-3'	5'-CTGGTGTGACTGTAAGTGGGG-3'
	*Adipoq*	5'-TGTTCCTCTTAATCCTGCCCA-3'	5'-CCAACCTGCACAAGTTCCCTT-3'
	*Ibsp*	5'-TAGTTCCGAAGAGGAGGGGG-3'	5'-CCCTGCTTTCTGCATCTCCA-3'
	*Cebpb*	5'-ACAAGCTGAGAGACGAGTACAAGA-3'	5'-GCAGCTGCTTGAACAAGTTCCG-3'
	*Klf5*	5'-AGGACTCATACGGGCGAGAA-3'	5'-ATGCACTGGAACGGCTTGG-3'
	*Runx2*	5'-GGT TAA TCT CCGCAG GTC ACT-3'	5'-CAC TGT GCT GAA GAG GCT GTT-3'
	*Alpl*	5'-CTGACTGACCCTTCGCTCTC-3'	5'-TGCTTGGCCTTACCCTCAT-3'

### Mitochondrial activity assays

Mitochondrial activity was determined by the assessment of OCR using Seahorse XFe24 Analyzer (Agilent Technologies, Santa Clara, CA, USA) as previously reported [47] and mitochondrial ΔΨm. with JC-1 (Thermo Fisher Scientific) staining according to the manufacturer’s protocol. For OCR assay, 2 × 10^4^ MSCs were seeded in a 24XF cell culture microplate, cultured for 8 h, and then replaced with Seahorse XF DMEM media in which contain 2% FBS, 2 mM glutamine and 1 g/L glucose with a 7.4 pH and incubated at 37 °C for extra 30 min. OCR was determined with a sequential treatment of 1 μM oligomycin, 2 μM carbonyl cyanide-p-trifluoromethoxyphenylhydrazone (FCCP) and rotenone combined with antimycin A during the analysis process. For mitochondrial ΔΨm. assay, 2 × 10^5^ MSCs were treated with 2.5 μM JC-1 and then incubated at 7 °C for 30 min. Fluorescence signals were captured by fluorescence microscopy or quantified by flow cytometry. Depolarized mitochondrial ΔΨm. was assessed by detecting green fluorescence signals while polarized mitochondrial ΔΨm. was assessed by detecting red fluorescence signals. Gating for fluorescent signals of green^+^/red^−^, green^+^/red^+^ and green^−^/red^+^ was also performed for frequency analyses of low, intermediate and high mitochondrial ΔΨm., respectively.

### Nicotinamide adenine dinucleotide (NAD^+^)/NADH assay

The ratio of cellular NAD^+^/NADH was assessed with colorimetric assay according to the manufacturer’s protocol (Abcam, Cambridge, UK). Briefly, NADH amount was first measured by heating at 60ºC for 30 min to decompose NAD^+^ with developer reaction for measurement at OD 450 nm, and then convert NAD^+^ to NADH for measurement of total NAD^+^ and NADH (NADt). NAD + /NADH ratio can be calculated by the formula: (NADt-NADH)/NADH.

### SIRT1 protein detection

Human BMMSCs were cultured in specific conditions for 8 h, with subsequent extraction of total cellular protein for assessment of SIRT1 expression with β-actin as an internal control by western blot analyses (all antibodies from Santa Cruz, CA, USA).

### In vivo mouse experimentation

Animal experimentation was conducted following approved protocols by the Institutional Animal Care and Use Committee of the National Taiwan University (No. 20170426). 6-week-old C57BL/6 male mice were given drinking water with added glucose prepared by adding 13 g D-glucose (Sigma) into 100 ml drinking water [48] for 1 day or 5 days. Nicotinamide mononucleotide (NMN) was also orally provided in drinking water with a concentration of 2 g/L [49], and sirtinol was intraperitoneally administered with a dose of 1 mg/kg/day [50]. Mice were euthanized on day 1 or day 5, with uncultured CD45- BM cells harvested for RNA extraction and assessment of gene expression as well as JC-1 staining for assessment of mitochondrial function.

### Statistical analysis

For comparisons between two groups, Student’s *t*-test was used, while for comparisons between multiple groups, ANOVA was used followed by Tukey test. A value of *p* < 0.05 was considered statistically significant. Analyses were performed using GraphPad Prism software (CA, USA) and data are shown as mean ± SD.

## Results

### Glucose-related metabolic pathways and mitochondria activity are integral to MSC lineage commitment, with HG alone without additional lineage induction able to shift human & murine MSCs to favor adipogenesis at the expense of osteogenesis

**Fig. 1:**
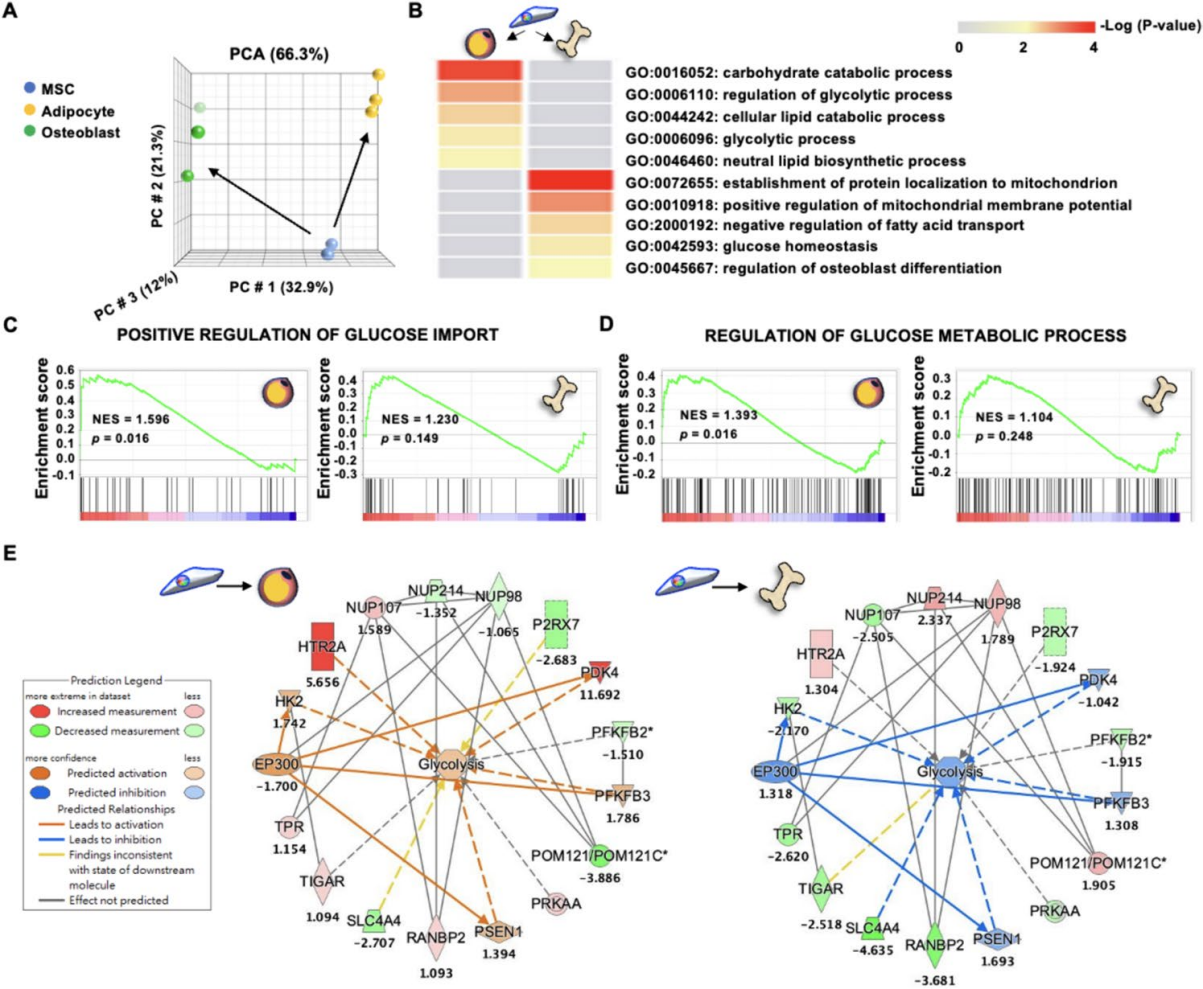
Mitochondrial and glucose homeostasis pathways were highly associated with osteogenesis, while glycolysis-related pathways were highly associated with adipogenesis. **A** Principal component analysis (PCA) of transcriptomic profiles of human bone marrow (BM) mesenchymal stem cells (MSCs; *n* = 3, GSE20631), adipocytes (*n* = 3, GSE45169), and osteoblasts (*n* = 3, GSE101140). **B** Metascape pathway analyses for enriched GO Biological Processes in adipocytes and osteoblasts compared to BMMSCs using transcriptomic data, with pathways colored according to *p* values. **C** Enrichment of “Positive regulation of glucose import” pathway as performed by Gene set enrichment analysis (GSEA) in adipocytes or osteoblasts compared to BMMSCs, with normalized enrichment score (NES) and *p* value labeled. **D** Enrichment of “Regulation of glucose metabolic process” pathway as performed by GSEA in adipocytes and osteoblasts compared to BMMSCs, with NES and *p* value labeled. **E** Prediction of mechanisms involved in “Glycolysis” as performed with the Molecular Activation Prediction (MAP) tool using transcriptomic data from adipocytes or osteoblasts compared to BMMSCs

**Fig. 2:**
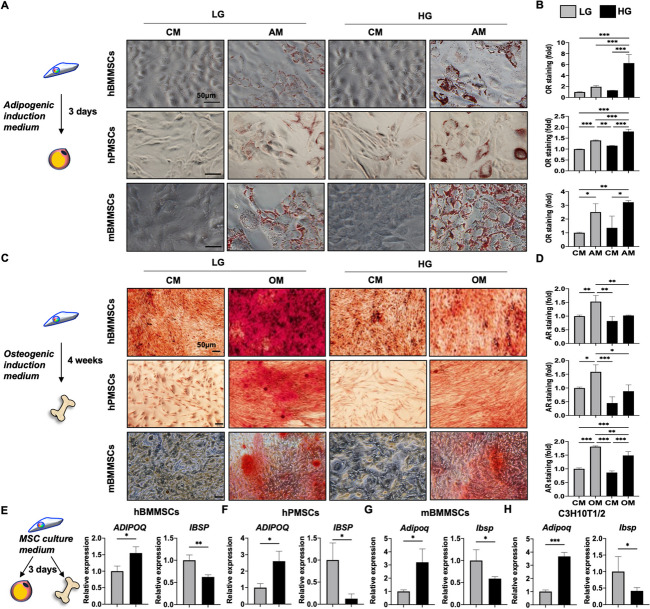
High glucose (HG) alone can commit human and murine MSCs towards adipogenesis while inhibiting osteogenesis. **A** and **B** Representative data and pooled data of lipid droplet accumulation as assessed in human BMMSCs (top panel), human placental MSCs (PMSCs; middle panel), or murine BMMSCs (bottom panel) by staining with Oil Red (OR). Scale bar, 50 μm. **C** and **D** Representative and pooled data of extracellular mineralization as assessed in human BMMSCs (top panel), human PMSCs (middle panel), or murine BMMSCs (bottom panel) by staining with Alizarin Red (AR). Scale bar, 50 μm. **E** and **F** qPCR analyses of functionally adipogenic and osteogenic genes, adiponectin (*ADIPOQ*) and bone sialoprotein (*IBSP*), in human BMMSCs (**E**) and PMSCs (**F**), treated with LG or HG for 3 days. **G** and **H** qPCR analyses of *Adipoq* and *Ibsp* in murine BMMSCs (**G**) and mouse C3H10T1/2 mesenchymal progenitor/stem cells (C3H; H), treated with LG or HG for 3 days. *N* = 3 for each group in all figures. Data are shown as mean ± SD. *, *p* < 0.05; **, *p* < 0.01; ***, *p* < 0.001

To assess the involvement of various energetic pathways in MSC lineage commitment towards adipogenesis or osteogenesis, we first performed PCA analysis of whole transcriptomic profiling of human BMMSCs, adipocytes and osteoblasts to ascertain that the 3 cell types have distinctive profiles (Fig. 1A). Bioinformatics analyses revealed a number of carbohydrate- and glycolysis-related metabolic pathways to be significantly enriched in adipocytes vs. BMMSCs, while mitochondria-related and glucose homeostasis pathways were significantly enriched in osteoblasts vs. BMMSC (Fig. 1B). GSEA also supported the importance of glucose-related processes in adipocytes but not osteoblasts vs. BMMSCs by significantly upregulating “Positive regulation of glucose import” pathway (Fig. 1C) and “Regulation of glucose metabolic process” (Fig. 1D). In addition, we used the MAP tool from IPA and found that the process of “Glycolysis” was upregulated in adipocytes but downregulated in osteoblasts when compared to BMMSCs (Fig. 1E), suggesting that this process is more critically involved in adipogenesis rather than osteogenesis. To determine whether changes in glucose levels could functionally affect MSC lineage commitment to adipogenesis or osteogenesis, we first assessed the gene expression of glucose transporters in both human and murine MSCs of two tissue sources. We found that expression levels of insulin-independent *GLUT1*/*Glut1* and *GLUT3*/*Glut3* were significantly higher than insulin-dependent *GLUT2*/*Glut2* and *GLUT4*/*Glut4* in primary human BMMSCs, human placental MSCs (PMSCs), and mouse BMMSCs regardless of treated glucose levels—either low glucose (LG; 5.5 mM) or HG (25 mM); similar findings were seen in the mouse C3H10T1/2 MSC line (C3H) which only expressed the insulin-independent *Glut1* (Figure S1). These data suggest that glucose uptake in MSCs is generally insulin-independent. In vitro functional assessment of adipogenesis was significantly increased in primary human and murine MSCs cultured in adipogenic medium (AM) containing HG compared to LG, as evidenced by Oil Red (OR) staining for oil droplets (Fig. 2A, B). However, osteogenic capacity of all three primary MSCs was significantly decreased in osteogenic medium (OM) with HG compared to LG, as assessed by Alizarin Red (AR) staining for calcium deposition (Figs. 2C, D). More surprisingly, even when MSCs were cultured in just control/expansion medium (CM) without any differentiation induction, addition of HG (HG-CM) increased expression levels of adiponectin (*ADIPQ*/*Adipoq*), an adipocyte lineage gene, in human BMMSCs and PMSCs as well as mouse BMMSCs and C3H, while simultaneously decreasing levels of bone sialoprotein (*IBSP*/*Ibsp*), an osteogenic lineage gene, in all four MSCs (Fig. 2E-H), suggesting a potential role for HG alone in driving MSC lineage specification towards adipogenesis over osteogenesis even without overt induction by lineage-specific differentiation medium. Collectively, these results suggest that HG alone can trigger lineage commitment of MSCs towards adipogenesis and away from osteogenesis.

### HG alone decreased mitochondrial activity and triggered MSC adipogenesis over osteogenesis within 8 h due to depletion of NAD+

**Fig. 3:**
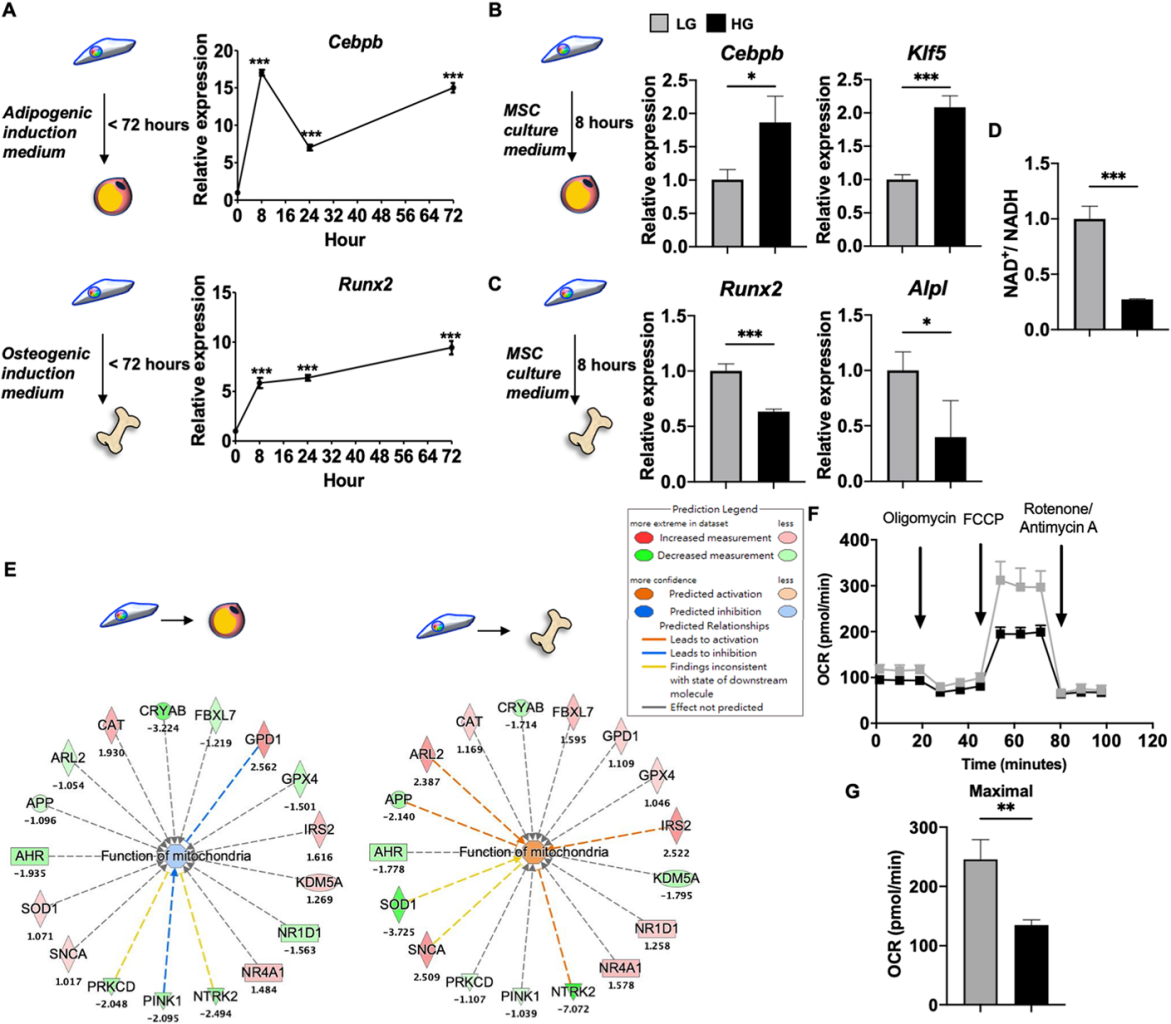
HG alone can trigger MSC adipogenesis over osteogenesis with depletion of nicotinamide adenine dinucleotide (NAD^+^) and depression of mitochondrial activity by 8 h. **A** qPCR analyses of early adipogenic and osteogenic genes, CCAAT/enhancer-binding protein beta (*Cebpb*) and Runt-related transcription factor (*Runx2*) as assessed in C3H with AM and OM, respectively. **B** qPCR analyses of early adipogenic genes, *Cebpb* and Kruppel-like factor 5 (*Klf5*) as assessed in C3H cells with CM containing LG or HG for 8 h. **C** qPCR analyses of early osteogenic genes, *Runx2* and alkaline phosphatase (*Alpl*) as assessed in C3H cells with CM containing LG or HG for 8 h. **D** Ratio of cellular NAD^+^ to NADH (NAD.^+^/NADH) as assessed in C3H with CM containing LG or HG for 8 h (*n* = 4 for each group). **E** MAP prediction of mechanisms involved in “Function of mitochondria” using transcriptomic data from human adipocytes (*n* = 3, GSE45169) or osteoblasts (*n* = 3, GSE101140) compared to BMMSCs (*n* = 3, GSE20631). **F** Oxygen consumption rates (OCRs) of C3H cultured in LG or HG conditions for 8 h as assessed by Seahorse assay. **G** Quantification of maximal OCRs in LG- or HG-cultured C3H. *N* = 3 for each group in all figures except D. Data are shown as mean ± SD. *, *p* < 0.05; **, *p* < 0.01; ***, *p* < 0.001

**Fig. 4:**
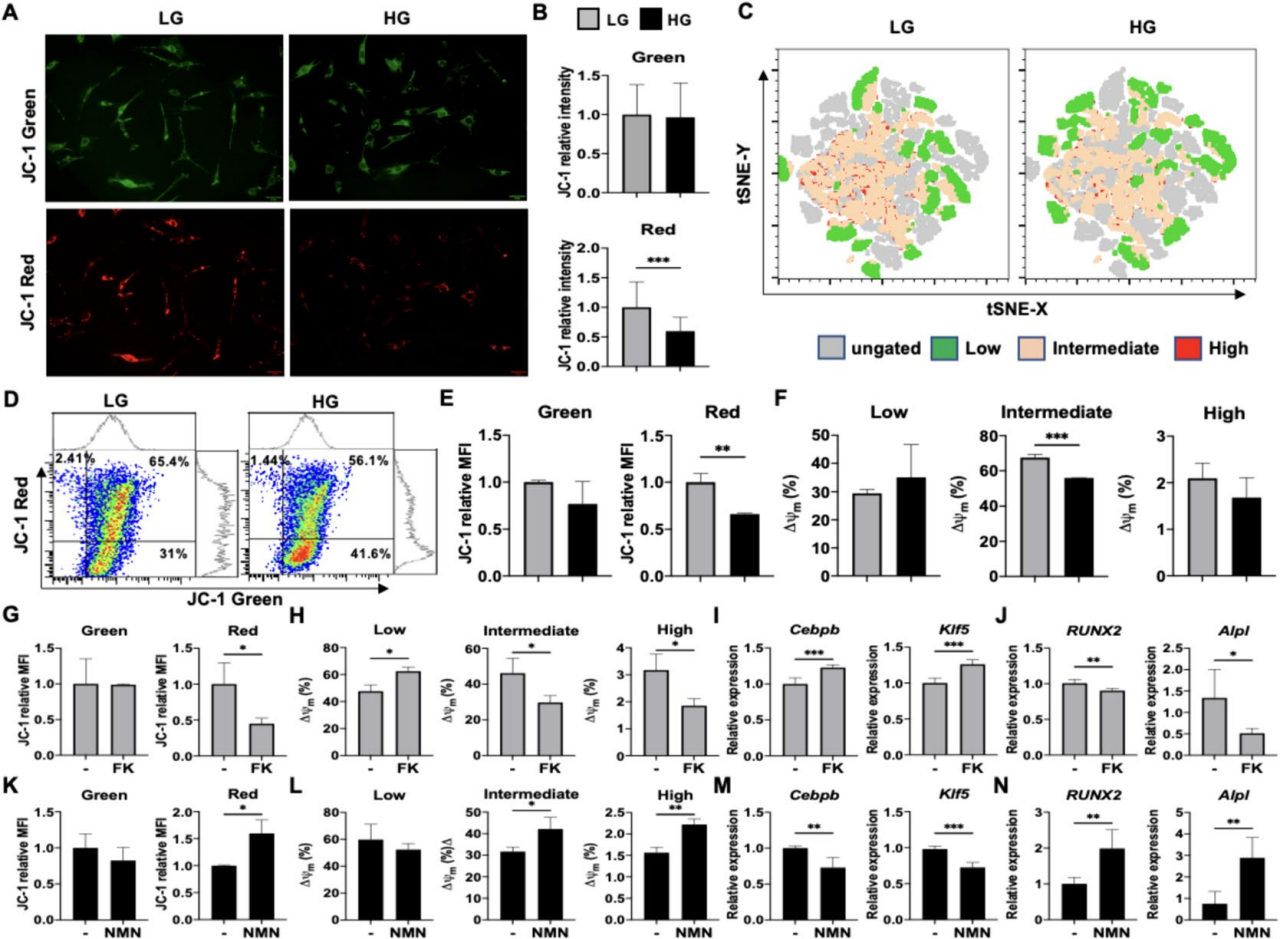
HG alone depress MSC osteogenesis and mitochondrial activity while enhancing adipogenesis, which are all reversed with nicotinamide mononucleotide (NMN) supplementation. **A** Representative data for mitochondrial activity of C3H as assessed with JC-1 staining by fluorescence microscopy at hour 8. Scale bar, 50 μm. **B** Pooled data for depolarized and polarized mitochondrial membrane potential (ΔΨm.) in C3H, as assessed by measuring fluorescence signals with Image J (three photos for each group with a total of 81 cells for LG and 83 cells for HG). **C** Population analyses for low, intermediate and high mitochondrial activity with PhenoGraft on the t-distributed stochastic neighbor embedding (t-SNE) map. C3H cells were cultured with LG or HG for 8 h, and then stained with JC-1, followed by flow cytometric analysis with t-SNE-based algorithm. **D** Representative data for frequency analyses of low, intermediate and high mitochondrial activity as assessed in C3H with CM containing LG or HG by flow cytometry at hour 8. **E** Pooled data of depolarized and polarized mitochondrial ΔΨm. as assessed in C3H with CM containing LG or HG by analyzing green intensity and red intensity, respectively. **F** Pooled data for frequency analyses of low, intermediate and high mitochondrial activity as assessed in C3H with CM containing LG or HG. **G** Pooled data of depolarized and polarized mitochondrial ΔΨm. as assessed in C3H with LG-CM in the absence or presence of FK866 (FK; 5 nM). **H** Pooled data for frequency analyses of low, intermediate and high mitochondrial activity as assessed in C3H with LG-CM in the absence or presence of FK. **I** and **J** qPCR analyses of *Cebpb* and *Klf5* (**I**) as well as *Runx2* and *Alpl* (J) as assessed in C3H with LG-CM in the absence or presence of FK. **K** Pooled data of depolarized and polarized mitochondrial ΔΨm. as assessed in C3H with HG-CM in the absence or presence of NMN (50 μM). **L** Pooled data for frequency analyses of low, intermediate and high mitochondrial activity as assessed in C3H with HG-CM in the absence or presence of NMN. (M and N) qPCR analyses of *Cebpb* and *Klf5* (**M**) as well as *Runx2* and *Alpl* (N) as assessed in C3H with HG-CM in the absence or presence of NMN. *N* = 3 for each group in all figures except B. Data are shown as mean ± SD. *, *p* < 0.05; **, *p* < 0.01; ***, *p* < 0.001

To further elucidate the mechanisms involved in triggering of MSC adipogenic over osteogenic commitment by HG alone, we first assessed the earliest time point at which the adipogenic and osteogenic master transcription factors CCAAT/enhancer-binding protein beta* (Cebpb)* and Runt-related transcription factor 2 (*Runx2*), respectively, are upregulated during differentiation into the respective lineages. To our surprise, we found that each master lineage transcription factor was rapidly induced in the respective media at just 8 h (Fig. 3A). To explore whether HG alone without differentiation induction could influence MSC lineage commitment also at this early time point, we profiled the 2 master transcription factors as well as 2 early markers for each lineage—Kruppel-like factor 5 (*Klf5*) for adipogenesis and alkaline phosphatase (*Alpl*) for osteogenesis—in C3H cultured in CM only with HG added. We found that expression levels of both *Cebpb* and *Klf5* were significantly upregulated in C3H with HG-CM at this early time point (Fig. 3B); conversely, expression of *Runx2* and *Alpl* were significantly downregulated at this time point in these same conditions (Fig. 3C). With the crucial role of NAD^+^ in metabolism, mitochondrial activity, as well as MSC osteogenesis via the NAD^+^-dependent deacetylase SIRT1, we assessed whether HG altered cellular levels of NAD^+^ and found that C3H cultured in HG-CM had a significantly decreased ratio of NAD^+^/NADH compared to cells cultured in standard LG-CM at 8 h (Fig. 3D). Bioinformatics analyses using the MAP tool predicted downregulation in the process of “Function of mitochondria” during MSC differentiation towards adipocytes but upregulation during differentiation towards osteoblasts (Fig. 3E). To confirm this prediction, we assessed the oxidative phosphorylation (OXPHOS) pathway using the Seahorse assay which revealed that MSCs cultured in HG demonstrated a significantly decreased maximal oxygen consumption rate (OCR; Fig. 3F, G, which suggests that the mitochondrial membrane potential (ΔΨm.)—an important parameter of mitochondrial function—was impaired by HG. To further validate these findings, we stained C3H cultured in HG-CM with a mitochondrial ΔΨm.-sensitive dye JC-1 and found at 8 h, these cells lost membrane potential compared to C3H cultured in LG-CM: similar intensity of green fluorescence representing depolarized mitochondrial ΔΨm. between LG- and HG-treated cells could be seen, but significantly lower intensity of red fluorescence representing polarized mitochondrial ΔΨm. was seen in HG- vs. LG-treated cells (Fig. 4A, B). To globally visualize the changes in mitochondrial ΔΨm. brought by HG, we performed t-distributed stochastic neighbor embedding (t-SNE) analyses and found that both intermediate and high ΔΨm. were more prominent in LG-treated cells than HG-treated cells (Fig. 4C). Verification with flow cytometry analyses (Fig. 4D-F) also demonstrated that HG similarly decreased the intensity of polarized mitochondrial ΔΨm.; further analyses also showed that HG significantly decreased mitochondria with intermediate ΔΨm. (represented by double positivity for red and green fluorescence), while increasing mitochondria with low ΔΨm. (green fluorescence positivity only) and decreasing mitochondria with high ΔΨm. (red fluorescence positivity only) albeit non-significantly.

To explore whether NAD^+^-mediated mitochondrial function is involved in regulating MSC lineage commitment, we inhibited NAD^+^ biosynthesis with FK866 (FK) in LG-CM-cultured C3H and at 8 h, and found that both the intensity of polarized mitochondrial ΔΨm. (Fig. 4G) as well as the frequency of mitochondria with either intermediate or high ΔΨm. were significantly decreased; the frequency of mitochondria with low ΔΨm. was significantly increased (Fig. 4H). Furthermore, analyses of lineage gene expression in FK-treated C3H demonstrated a shift from osteogenesis to adipogenesis, as evident by significant upregulation of *Cebpb* and *Klf5* with concomitant downregulation of *Runx2* and *Alpl* (Fig. 4I, J). Conversely, supplementation with nicotinamide mononucleotide (NMN), a precursor of NAD^+^ [31, 51], to C3H cultured in HG-CM resulted in an increase in NAD^+^ levels in just 8 h which reversed mitochondrial ΔΨm. and the frequencies of mitochondria with intermediate and high ΔΨm. (Fig. 4K, L); moreover, lineage commitment was shifted back to osteogenesis and away from adipogenesis as evidenced by significant downregulation of *Cebpb* and *Klf5* with significant upregulation of *Runx2* and *Alpl* simultaneously (Fig. 4M, N). To further confirm the role of NAD^+^-mediated mitochondrial metabolism in MSC lineage commitment, we cultured C3H in CM containing galactose (Gal) which diverts cells from glycolysis to mitochondrial metabolism thereby rapidly increasing cellular NAD^+^ within a few hours (Figure S2A) [52]. Mitochondrial parameters in Gal-CM-cultured C3H demonstrated significantly increased ΔΨm. (Figures S2B, C), with the frequency of mitochondria with low ΔΨm. significantly decreased while frequency of mitochondria with intermediate ΔΨm. was significantly increased (Figure S2D). Treatment with Gal also shifted C3H lineage commitment towards osteogenesis, with significant downregulation of *Cebpb* and *Klf5* alongside significant upregulation of *Runx2* and *Alpl* at 8 h’ time (Figures S2E-F). HG in vitro conditions have long been known to promote oxidative stress in numerous cell types [53–55], therefore to evaluate whether oxidative stress was involved in this rapid lineage commitment shift in MSCs, we assessed levels of reactive oxidative species (ROS) in LG vs. HG-treated MSCs. We found that ROS levels were similar in LG- and HG-treated MSCs up until 24 h of time and not significantly changed until 48 h after (Figure S2G, H), which is much later than the depletion of NAD^+^ with HG at 8 h. Collectively, these findings demonstrate that HG conditions rapidly reduce cellular NAD^+^ levels, leading to diminished mitochondrial function and a shift in MSC lineage commitment from adipogenesis to osteogenesis within 8 h. These consequences of HG can be reversed by supplementation with NMN/NAD^+^ or inhibition of NAD^+^ depletion.

### HG alone rapidly downregulates SIRT1 expression and functions to suppress mitochondrial activity as well as MSC osteogenesis via NAD^+^ depletion

**Fig. 5:**
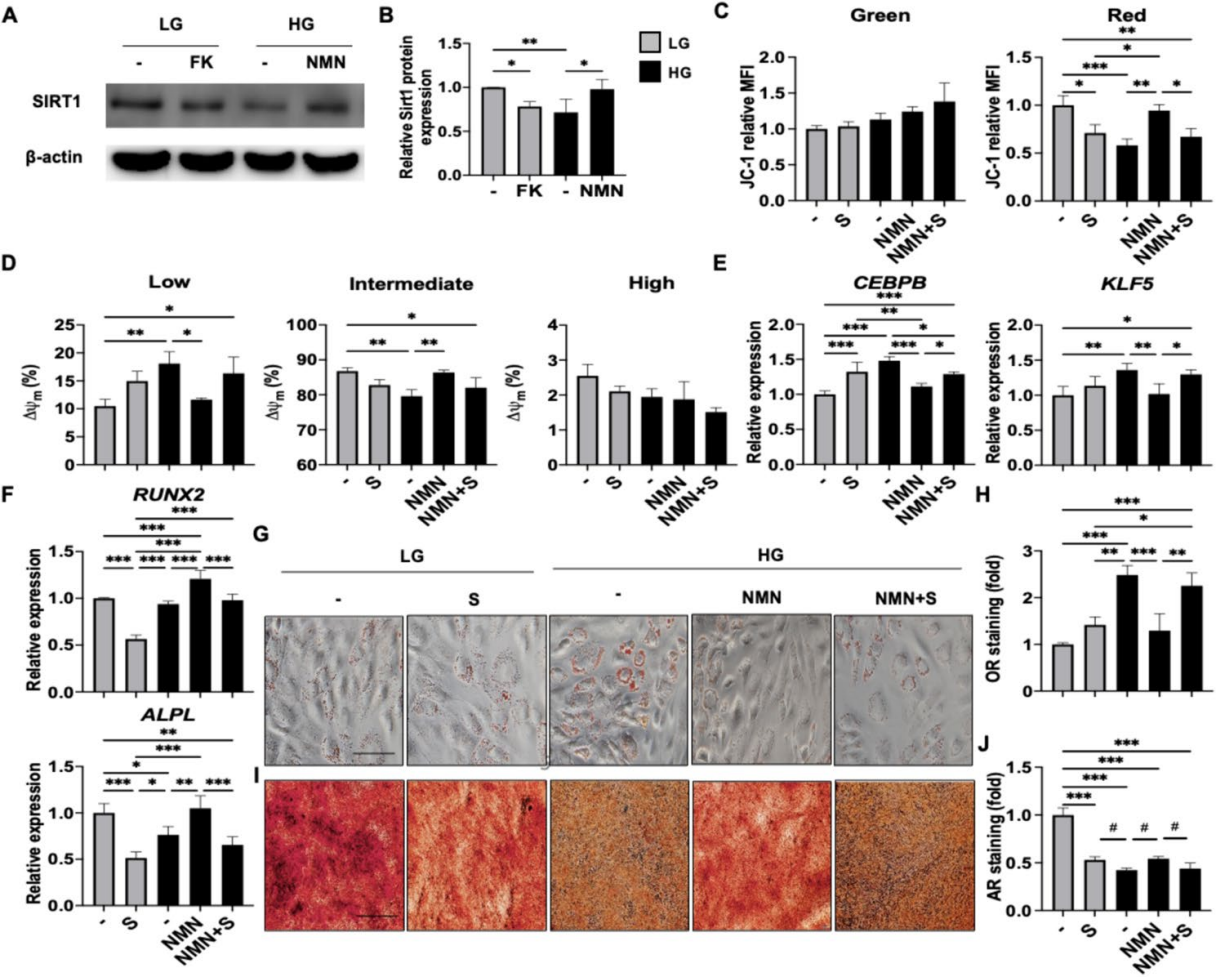
HG alone rapidly downregulates Sirtuin (SIRT) 1 expression and function to suppresses mitochondrial activity as well as MSC osteogenesis via NAD^+^ depletion. **A** & **B** Representative and pooled data for expression levels of SIRT1 protein in human BMMSCs cultured in LG-CM or HG-CM for 8 h as assessed by western blot, respectively (*n* = 4 for each group). **C** Pooled data of depolarized and polarized mitochondrial ΔΨm. as assessed in human BMMSCs treated with LG-CM in the absence or presence of sirtinol (S; 20 μM), or HG-CM in the absence or presence of NMN or NMN combined with S for 8 h (*n* = 3 for each group). **D** Pooled data for frequency analyses of low, intermediate and high mitochondrial activity as assessed in human BMMSCs treated with LG-CM in the absence or presence of S, or HG-CM in the absence or presence of NMN or NMN combined with S for 8 h (*n* = 3 for each group). (E and F) qPCR analyses of *CEBPB* and *KLF5* (**E**) as well as *RUNX2* and *ALPL* (**F**) as assessed in human BMMSCs treated with LG-CM in the absence or presence of S, or HG-CM in the absence or presence of NMN or NMN combined with S for 8 h (*n* = 4 for each group). **G** and **H** Representative and pooled data (*n* = 3 for each group), respectively, for oil drop accumulation in human BMMSCs as assessed with OR staining. Scale bar, 50 μm. **I** and **J** Representative and pooled data (*n* = 3 for each group), respectively, for calcium deposition in human BMMSCs as assessed with AR staining. Scale bar, 50 μm. Data are shown as mean ± SD. *, *p* < 0.05; **, *p* < 0.01; ***, *p* < 0.001. #, *p* < 0.05 for comparison excluding LG-OM (1.^st^ bar)

SIRT1 activity is NAD^+^-dependent, which uniquely confers a dual role for this molecule in MSCs: modulating mitochondrial function as well as osteogenic lineage commitment [56]. Surprisingly, however, understanding of how excess levels of simple sugars can affect these dual aspects of SIRT1’s actions in MSCs remain unclear. To explore whether the NAD^+^/SIRT1 axis is involved in HG-mediated MSC mitochondrial metabolic changes and adipogenic/osteogenic lineage commitment, we first assessed SIRT1 protein expression at 8 h and found that protein levels were significantly decreased in human BMMSCs cultured in HG-CM compared to cells cultured in LG-CM (Fig. 5A, B). Moreover, inhibition of NAD^+^ biosynthesis with FK significantly decreased SIRT1 protein levels in LG-CM-cultured human BMMSCs, whereas NMN supplementation significantly increased SIRT1 levels in HG-CM-cultured cells. Higher NAD^+^ levels have been shown to enhance SIRT1 expression in a number of studies [57, 58], and the mechanism appears to be mainly through protein stabilization including NAD^+^-mediated downregulation of JNK phosphorylation [59, 60] which we also found to be true (Figure S3A-D).

To assess the role of SIRT1 in modulating MSC mitochondrial activity, we treated LG-CM-cultured human BMMSCs with the SIRT1 inhibitor sirtinol (S) for 8 h which significantly decreased polarized mitochondrial ΔΨm. to levels similar to HG-CM-cultured cells; this could be rescued with NMN supplementation but not if SIRT1 activity was blocked (Figs. 5C & S3E-F). Flow cytometric analyses also demonstrated that inhibition of SIRT activity in LG-CM-cultured human BMMSCs increased the frequency of mitochondria with low ΔΨm., which were significantly decreased in HG-CM-culture cells supplemented with NMN; further inhibition of SIRT1 activity reversed this effect (Figs. 5D & S3G). The inverse trend was seen for mitochondria with intermediate ΔΨm., in which SIRT1 inhibition in LG-CM-cultured human BMMSCs decreased frequencies; in cells cultured in HG-CM, frequencies of such mitochondria were significantly increased after NMN supplementation and decreased after simultaneous SIRT1 inhibition (Figs. 5D & S3H-I). At 8 h’ time, adipogenic genes *CEBPB* and *KLF5* were upregulated whereas osteogenic genes *RUNX2* and *ALPL* were downregulated in LG-CM-cultured human BMMSCs, as well as cells cultured in HG-CM with NMN supplementation after SIRT1 inhibition (Figs. 5E-F & S3J-M). Functional differentiation assays validated the lineage-specific gene expression patterns (Figs. 5G-J & S3N-O): addition of HG to AM resulted in a doubling of oil droplet formation in human BMMSCs compared to LG-AM induction; conversely, HG-OM culture resulted in a more than 50% decrease in mineralization compared to LG-OM. Interestingly, SIRT1 inhibition in LG conditions increased oil droplet accumulation in AM-cultured human BMMSCs by over 40%, in OM-culture cells a more severe reduction of extracellular mineralization by nearly half was seen, and while NMN supplementation in HG-AM significantly decreased oil droplet formation, simultaneous inhibition of SIRT1 abrogated this effect; the converse was seen in HG-OM cultured human BMMSCs, with NMN supplementation resulting in a significant increase of mineralization compared to baseline HG-OM levels, which was largely abrogated when SIRT1 was inhibited at the same time. Interestingly, increasing *SIRT1* at the level of gene expression can also achieve similar results (Figure S3P-T), underscoring the critical role of SIRT1 in MSC lineage commitment. These findings demonstrate that LG conditions promote MSC osteogenesis as well as mitochondrial function through upregulation of the NAD^+^/SIRT1 axis, while HG conditions promote MSC adipogenic commitment and decrease mitochondrial metabolism by downregulating this axis within 8 h.

### In vivo HG intake rapidly downregulates mitochondrial activity and triggers adipogenic over osteogenic commitment in CD45^−^ BM cells that can be rapidly and strongly reversed with oral NMN supplementation in a SIRT1-dependent fashion

**Fig. 6:**
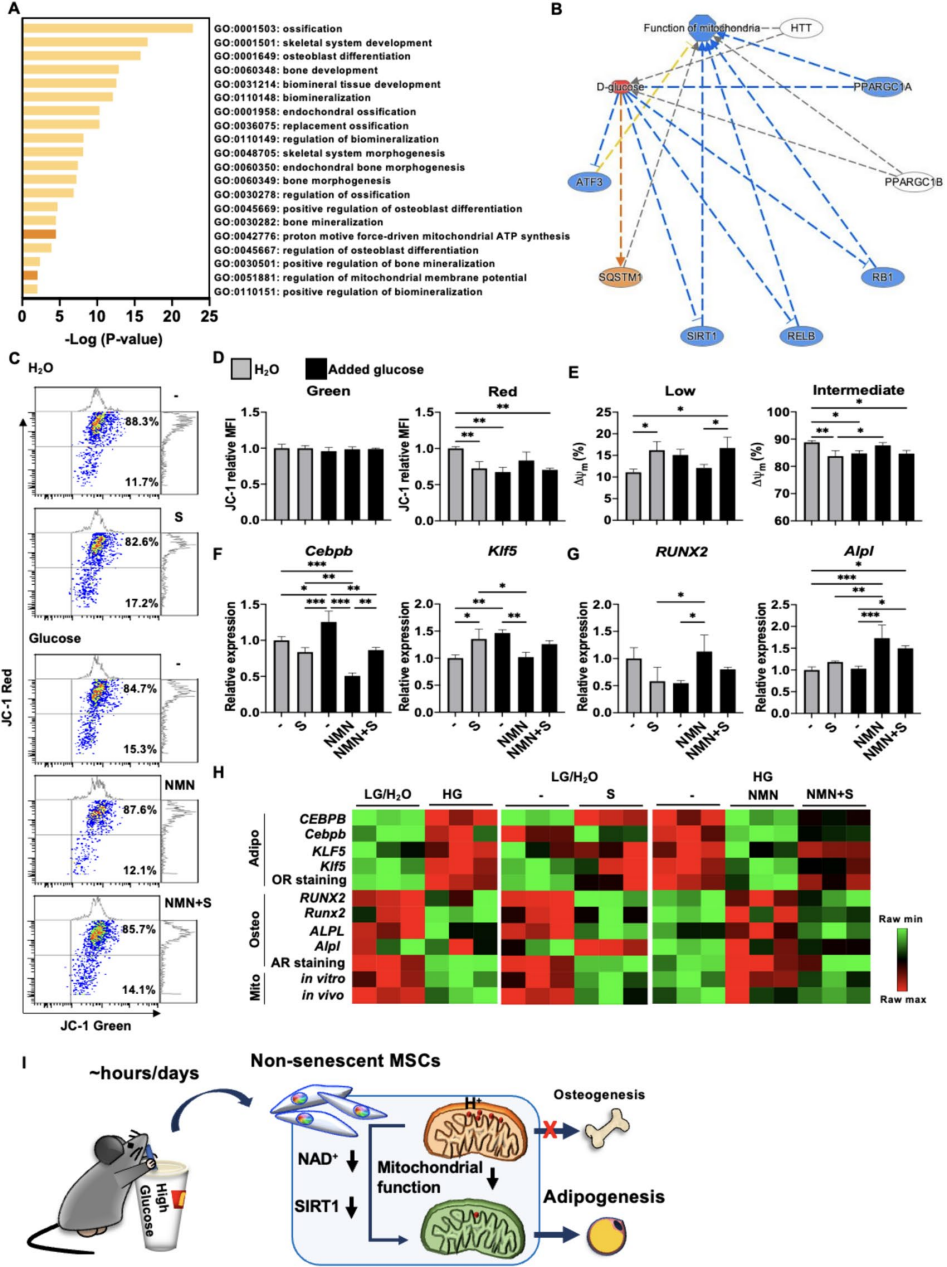
In vivo HG intake rapidly downregulates mitochondrial activity and triggers adipogenic over osteogenic commitment in CD45^−^ BM cells that can be rapidly and strongly reversed with oral NMN supplementation in a SIRT1-dependent fashion. **A** Enrichment of bone- and mitochondria-related pathways as assessed in single-cell RNA transcriptomics of uncultured BM CD45^−^ osteolineage cells vs. MSCs harvested from young adult mice (NCBI-GEO database: GSE128423) using Metascape analysis. **B** Pathway analysis on the process of “function of mitochondria” involving transcription regulators with treatment of high levels of glucose as performed by MAP tool in IPA. The MAP tool showed that “function of mitochondria” is downregulated (blue) through positively regulated (orange line), negatively regulated (blue lines), and unpredicted (yellow line) downstream molecules after treatment of high levels of glucose (red). **C** Representative data for frequency analyses of low and intermediate mitochondrial activity as assessed in uncultured CD45^−^ BM cells of wildtype young adult C57BL/6 mice fed normal drinking water combined with intraperitoneal (i.p.) administration of S or not, or given drinking water with additional glucose with or without NMN, or NMN combined with i.p. injection of S. **D** Pooled data of depolarized and polarized mitochondrial ΔΨm. as assessed in CD45^−^ BM cells. **E** Pooled data for frequency analyses of low and intermediate mitochondrial activity as assessed in uncultured CD45^−^ BM cells. **F** and **G** qPCR analyses of *Cebpb* and *Klf5* (**F**) as well as *Runx2* and *Alpl* (**G**) in BM cells. *N* = 3 for each group in all figures. Data are shown as mean ± SD. *, *p* < 0.05; **, *p* < 0.01; ***, *p* < 0.001. **H** Heatmap plots as generated comparing relative expression levels of *CEBPB*/*Cebpb*, *KLF5*/*Klf5*, OR staining, *RUNX2*/*Runx2*, *ALPL*/*Alpl*, AR staining as well as in vitro and in vivo mitochondrial activities between LG and HG groups in vitro or H_2_O and glucose groups in vivo. (I) Summary: HG intake for a few hours or days rapidly depresses mitochondrial activity and osteogenesis while strongly promoting adipogenesis via the NAD^+^/SIRT1 axis in non-senescent MSCs, with rescue of both mitochondrial activity and osteogenesis possible through NMN supplementation

To investigate the translational relevance of the in vitro results of HG conditions on MSC mitochondrial metabolism and lineage commitment, we first performed bioinformatics analyses to assess relevant pathways in single-cell RNA transcriptomics of uncultured CD45^−^ BM osteolineage cells vs. MSCs harvested from young adult mice. We found that pathways involving bone formation and mitochondrial activities including ΔΨm. and ATP synthesis were enriched (Fig. 6A); moreover, treatment with high levels of glucose was predicted to downregulate mitochondrial functions via multiple transcription regulators including SIRT1 (Fig. 6B). To functionally validate the role of SIRT1 and NAD^+^ in HG-downregulated mitochondrial metabolism and MSC osteogenesis in vivo, we fed wildtype young adult mice drinking water with additional glucose and assessed the mitochondrial function as well as lineage-specific genes for adipogenesis and osteogenesis in uncultured CD45^−^ BM cells which contain the MSC population [61, 62]. Acutely, blood glucose levels were significantly increased in mice receiving glucose-supplemented drinking water compared to those receiving normal water at 4 h. However, the blood glucose levels were similar between the two groups on days 2 and 6 (Figure S4A). These results are in line with previous studies which show that in normal mice, glucose intake rapidly elevates blood glucose levels within hours but can be restored to normal levels thereafter [63, 64]. We found that CD45^−^ BM cells isolated from mice after 1 day or 5 days of HG intake lost mitochondrial ΔΨm. significantly (Figs. 6C, D & S4B-C); and frequencies of mitochondria with low ΔΨm. were significantly increased whereas mitochondria with intermediate ΔΨm. was significantly decreased at both time points as well (Figs. 6E & S4D-E). In mice drinking normal water, SIRT1 inhibition for 1 day significantly decreased the intensity of mitochondrial ΔΨm. as well as frequency of mitochondria with intermediate ΔΨm., while significantly increasing mitochondria with low ΔΨm. in CD45^−^ BM cells. Conversely, in mice drinking HG-water, NMN supplementation for 1 day increased the frequency of mitochondria with intermediate ΔΨm. in CD45^−^ BM cells which could be significantly reversed with SIRT1 inhibition; however, at day 5 NMN supplementation did not alter mitochondrial function regardless of whether additional glucose was given or not (Figures S4F-G). Interestingly, lineage commitment towards adipogenesis and away from osteogenesis occurred rapidly after 1 day of HG intake, with significantly upregulated levels of *Cebpb* in CD45^−^ BM cells regardless of whether NAD^+^ or SIRT1 was modulated (Figures S4H-I); no changes in levels of osteogenic genes were seen. But by 5 days of HG intake, *Runx2* expression was significantly downregulated, and both *Cebpb* and *Klf5* were upregulated in CD45^−^ BM cells; moreover, at this time point, NMN supplementation during HG intake significantly decreased both *Cebpb* and *Klf5* while increasing *Runx2* and *Alpl* expression (Figs. 6F, G and S4J-M). SIRT1 inhibition at this time point in normal water intake conditions had a mixed effect on both lineages, significantly decreasing *Cebpb* levels but significantly increasing *Klf5* levels, while decreasing *Runx2* levels and minimally affecting *Alpl*; however, SIRT1 inhibition under NMN supplemented-HG conditions increased levels of *Cebpb* and *Klf5* while decreasing levels of *Runx2* and *Alpl* compared to NMN supplementation alone. These findings demonstrate that while NAD^+^ depletion due to HG intake can be strongly reversed with oral NMN supplementation, SIRT1 activity is required. Interestingly, the mitochondrial ΔΨm. decline seen with HG intake for 1 day can be restored after reversion to regular water for 5 days, but the lineage commitment shift towards adipogenesis/away from osteogenesis could not be restored (Figure S5), implicating the tremendous insult that HG has on MSC osteogenesis. Moreover, HG intake could have consequences even for end-differentiated adipocytes and osteoblasts; when we lengthened the experiment out to 3-week’s time, we found that marrow adipocytes [65, 66] were significantly increased while marrow osteoblasts [67, 68] were significantly decreased in mice drinking HG- vs. regular water (Figure S6). These findings implicate that longer term HG intake may have consequences on more end-differentiated adipocytes and osteoblasts as well. To visually summarize all assessed assays, we utilized heatmap analysis to demonstrate the clear shift in MSC differentiation potential from osteogenesis to adipogenesis as well as mitochondrial functional decline under HG treatment whether in in vitro human or mouse, or in vivo mouse systems (Fig. 6H). Interestingly, there are only 2 discrepant trends seen in the gene expression data of murine *Cebpb* and *Alpl* mainly under normal/LG conditions; both genes are known to involved in the regulation of other stem cell compartments and processes [69–71], which was evident in the in vivo/murine setting of evaluating an entire organ/tissue but not the in vitro/human setting of evaluating only MSCs. Overall, these results demonstrate that in vivo HG intake rapidly downregulates MSC compartment mitochondrial activity as well as strongly modulates lineage commitment towards adipogenesis via the NAD^+^/SIRT1 axis within 1 to 5 days, with rescue of both mitochondrial activity and osteogenesis possible using oral NMN supplementation which is dependent on SIRT1 activity. Our study provide evidence that HG alone is enough to detrimentally alter young, non-senescent MSC mitochondrial function and osteogenesis to favor adipogenesis within hours in vitro and days in vivo through NAD^+^ depletion and SIRT1 activity (Fig. 6I).

## Discussion

Because over 90% of bone mass is acquired by late adolescence/early adulthood, interventions to significantly improve bone health and decrease OP risk would have the most impact when implemented in these youthful populations [20–22]. Our findings have strong translational implications since we found HG alone-mediated skewing of differentiation from osteogenesis to adipogenesis occurring in non-senescent MSCs in vitro and young mice in vivo. The gain of adipogenic commitment capacity at the expense of osteogenesis in senescent human MSCs is well documented, both in chronological as well as replicative senescence [13, 15–18]. The decreased osteogenesis in aged BM and MSCs has been attributed to decreased SIRT1 levels and/or activity—largely due to senescence-related increased oxidative stress [72, 73] and lower NAD^+^ levels [37, 38]. In contrast to these studies, we found that even in young, non-senescent MSCs, HG-mediated NAD^+^ depletion depressed mitochondrial and SIRT1 function to increase MSC adipogenic commitment at the expense of osteogenic commitment in both in vitro and in vivo settings. Moreover, our data implicates that while the NAD^+^ depletion and subsequent detrimental impact on osteogenic commitment caused by a shorter duration of HG conditions (less than 7 days: Figs. 5A-F and 6C-G) can be readily reversed with NMN supplementation, long-term HG conditions (more than 7 days: Figs. 2C, D, 5I, J, S5, and S6) may have a more profound adverse effect on MSC osteogenesis, which has been overwhelmingly found to be more fragile than adipogenesis [74, 75]. Our current data may offer an explanation with regards to the low bone mass seen in teenagers and young adults with the highest intake of simple sugars [24, 26]. Modifiable factors to enhance bone mass are few, with dietary habits being one of such few factors, but while excessive simple sugar intake is well known to increase cardiometabolic disease burden, its link to bone health has not been consistently demonstrated. We found MSCs to express insulin-independent transporters similar to their progeny the osteoblast [30], implicating that MSCs can respond directly to surrounding glucose levels. Our findings may help clarify the correlation of ever higher simple sugar consumption globally in younger and younger age groups [76, 77] to the trend of low bone mass in this age group [21, 22]. Disturbingly, our findings here suggest a possible “double blow” of simple sugar overconsumption on bone health in non-aged, more youthful stages of life—e.g. during adolescence/early adulthood—by directly decreasing the osteogenic capacity of MSCs at the only window of time when peak bone mass can be acquired.

Our report further contributes to the well-documented pro-osteogenic activity of SIRT1 [78, 79]. Through its deacetylase activity and also as a co-factor to a number of developmentally important transcription factors including β-catenin and Foxo3a, we and others have demonstrated that SIRT1 promotes osteogenesis in MSCs and osteoprogenitors by directing increasing expression of RUNX2 and downstream genes including ALPL [35, 36, 80]. More recently, SIRT1-mediated deacetylation of RUNX2 was found to enhance its transcriptional activity [81]. Additionally, likely consequent to the often observed inverse relationship between osteogenesis and adipogenesis, SIRT1 also has been shown to decrease functional MSC adipogenesis [82] as well as *CEBPB* [83] and *KLF5* expression [36]. Collectively, our current findings and these previous reports show that SIRT1 agonism, including via NAD supplementation, strongly shifts MSC lineage commitment towards osteogenesis and away from adipogenesis.

The rapidity and singularity of HG alone in adversely modulating both MSC mitochondrial function and lineage commitment across tissue sources and species of MSCs without the need for lineage-inducing environments was impressive. We found the single molecular crux modulating these functional outcomes to be NAD^+^, a molecule central to mitochondrial activity as well as SIRT1 function which modulates the osteogenic transcriptional program through Runx2, the master transcription factor for osteogenesis [35, 84]. When glucose is the predominant substrate, glycolysis is favored over mitochondrial oxidative phosphorylation with consequent depletion of NAD^+^, which in turn alters the NAD^+^/NADH ratio [32, 85]. This resultant sub-optimal NAD^+^/NADH ratio not only compromises mitochondrial oxidative phosphorylation efficiency and mitochondrial membrane potential, but may even lead to cell death [85]. HG can also have direct detrimental effects on mitochondrial function and membrane potential by promoting mitochondrial fission and fragmentation, leading to impaired mitochondrial function and decreased membrane potential ultimately reducing ATP production [86], as well as activating stress signaling pathways such as the NF-κB pathway to contribute to mitochondrial damage [87]. While known for providing energy needed for cell growth and proliferation, the mitochondria within stem cells is increasingly found to be involved in determining cell fate; however, its role in MSC lineage commitment has been less explored [88]. Moreover, while perturbations—including those caused by dietary changes—to NAD^+^ levels clearly affect mitochondrial function [89, 90], these important energetics and metabolic aspect of this axis have surprisingly been little explored in MSC biology, with an early study reported mitochondrial membrane potential increasing in in vitro cultured osteoblasts during differentiation into a more mature phenotype [91], and a recent study showed stronger mitochondrial activity in non-senescent MSCs [92]. As complex as mitochondrial molecular mechanisms and functions are, the fact that our findings converge on NAD^+^, a single molecule highly sensitive to metabolic changes, helps to explain the rapidity of functional outcomes as well as broad effects of HG alone on both MSC mitochondrial function and lineage commitment. Indeed, the reversal of NAD^+^ depletion and its adverse consequences with NMN supplementation also was broad and rapid, occurring within hours/days. Our data strongly implicate that in MSCs, the two functional events of lineage commitment and mitochondrial function are inextricably linked, with stronger mitochondrial function linked to osteogenic commitment controlled by NAD^+^ levels.

Encouragingly, we found that NAD^+^ depletion due to HG intake can be rapidly reversed with NMN supplementation in these youthful/non-senescent systems in a SIRT1-dependent fashion, reversing MSC adipogenic commitment back towards osteogenesis and improving mitochondrial function as well. NAD^+^ biology and supplementation have increasingly been considered to be at the center of numerous age-related functional decline and pathologies, with improving mitochondrial function a commonality in these various aging and disease processes [51]. The ability of NMN supplementation to reverse the anti-osteogenic/pro-adipogenic effects of HG intake also provides an additional weapon with fewer adverse effects and possibly better compliance in the armamentarium against OP and poor bone health. Interestingly, in the in vivo model, adipogenic genes were upregulated more rapidly than osteogenic genes perhaps reflecting the well-known in vitro time course of MSC adipogenesis being much more rapid and achieved in days compared to osteogenesis, which can require up to several weeks [39, 40]. Nevertheless, the overall rapid trend of pro-adipogenic/anti-osteogenic impact of HG intake was seen in vivo as well, with NMN supplementation also able to reverse these changes also within days. The rapidity of NMN-mediated functional reversal in our study is likely due to the youthful/non-senescent MSCs and mice we tested, since recent studies in aged mice demonstrate the need for several weeks to months of supplementation for reversal to occur [90, 93].

## Conclusion

Our findings are the first to demonstrate the significant and detrimental impact of a single dietary factor alone on MSC osteogenic capacity in youthful settings. Given the few anabolic therapeutic options, the outsize contribution of OP to global disease disability, and that 90% of peak bone mass is acquired by late adolescence/early adulthood, our data strongly implicate that excessive simple sugar consumption in youthful populations may have far-reaching consequences on bone health. This study also demonstrates NAD^+^ not only as the crucial molecule linking HG intake to both MSC mitochondrial activity and osteogenic commitment in non-senescent settings, but also as a probable therapeutic target for improving bone health irrespective of age in our current global environment of sugar overconsumption.

### Supplementary Information

Supplementary Material 1.

## Data Availability

The microarray datasets used in this study can be accessed at NCBI-GEO: GSE20631 (https://www.ncbi.nlm.nih.gov/geo/query/acc.cgi?acc=GSE20631), GSE45169 (https://www.ncbi.nlm.nih.gov/geo/query/acc.cgi?acc=GSE45169), GSE101140 (https://www.ncbi.nlm.nih.gov/geo/query/acc.cgi?acc=GSE101140), and GSE128423 (https://www.ncbi.nlm.nih.gov/geo/query/acc.cgi?acc=GSE128423).
