# Correction: Excess glucose alone depress young mesenchymal stromal/stem cell osteogenesis and mitochondria activity within hours/days via NAD^+^/ SIRT1 axis

**DOI:** 10.1186/s12929-024-01046-1

**Published:** 2024-06-07

**Authors:** B. Linju Yen, Li‑Tzu Wang, Hsiu‑Huang Wang, Chin‑Pao Hung, Pei‑Ju Hsu, Chia‑Chi Chang, Chien‑Yu Liao, Huey‑Kang Sytwu, Men‑Luh Yen

**Affiliations:** 1 Regenerative Medicine Research Group, Institute of Cellular & System Medicine, National Health Research Institutes (NHRI), No.35, Keyan Road, Zhunan, 35053 Taiwan; 2 Department of Obstetrics & Gynecology, National Taiwan, University (NTU) Hospital & College of Medicine, NTU, No.1, Section 1, Jen‑Ai Road, Taipei, 10051 Taiwan; 3 School of Medical Laboratory Science and Biotechnology, College of Medical Science and Technology, Taipei Medical University, No. 250, Wuxing Street, Taipei, 11042 Taiwan; 4 Ph.D. Program in Medical Biotechnology, College of Medical Science and Technology, Taipei Medical, University, No.250, Wuxing Street, Taipei, 11042 Taiwan; 5 Graduate Institute of Life Sciences, National Defense Medical Center (NDMC), No.161, Section Minquan East Road, Taipei, 11490 Taiwan; 6 National Institute of Infectious, Diseases & Vaccinology, NHRI, No.35, Keyan Road, Zhunan, 35053 Taiwan; 7 Graduate Institute of Microbiology & Immunology, NDMC, No.161, Section 6, Minquan East Road, Taipei, 11490 Taiwan

**Correction: J Biomed Sci 31, 49 (2024)**

**https://doi.org/10.1186/s12929-024-01039-0**

After publication of the article [1], it was brought to our attention that:

Figures 2 and Fig. 3 in the original paper still have revision red lettering on some legends. The all-black lettering figures are shown below:

Figure 2.
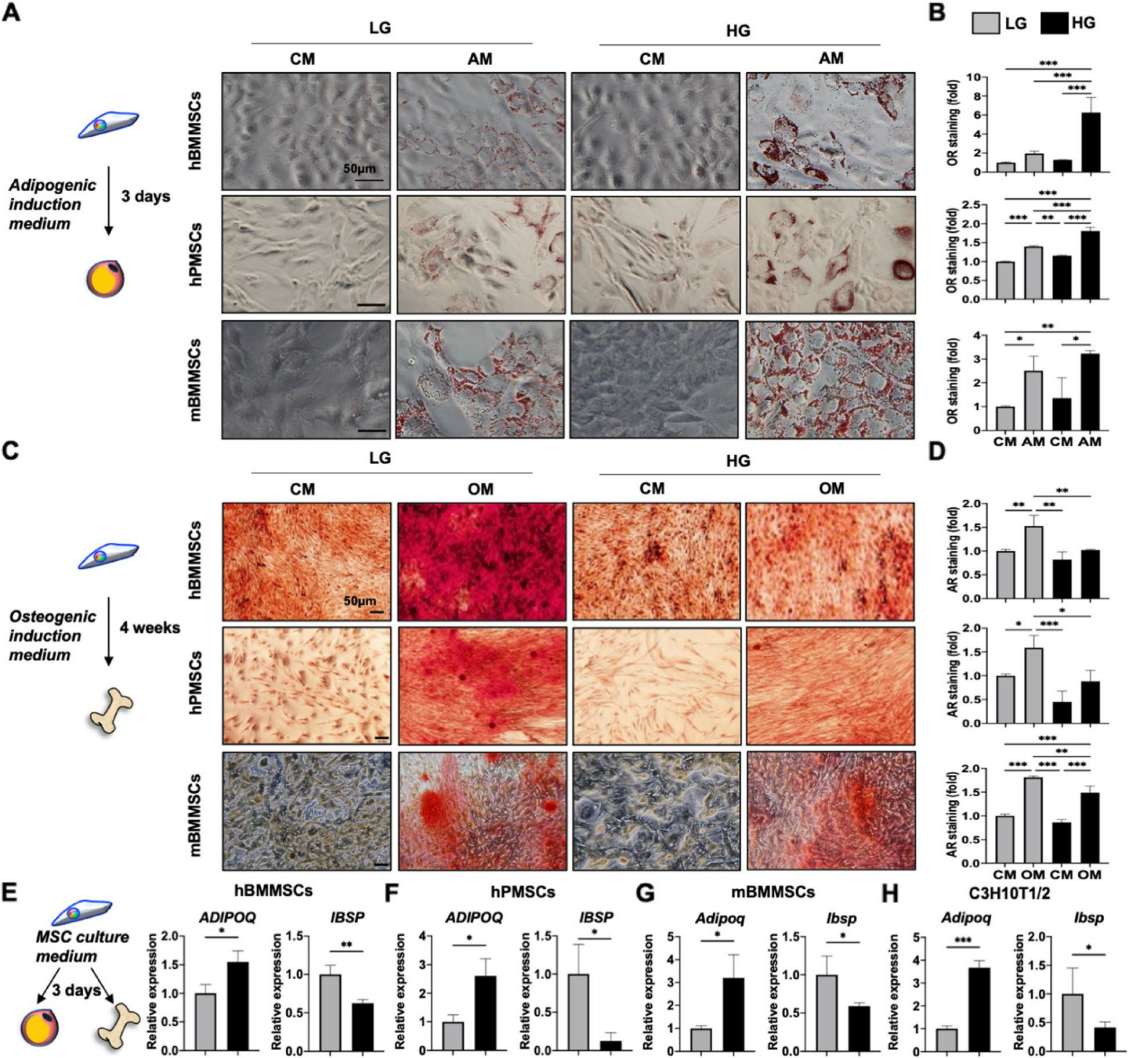


Figure 3.
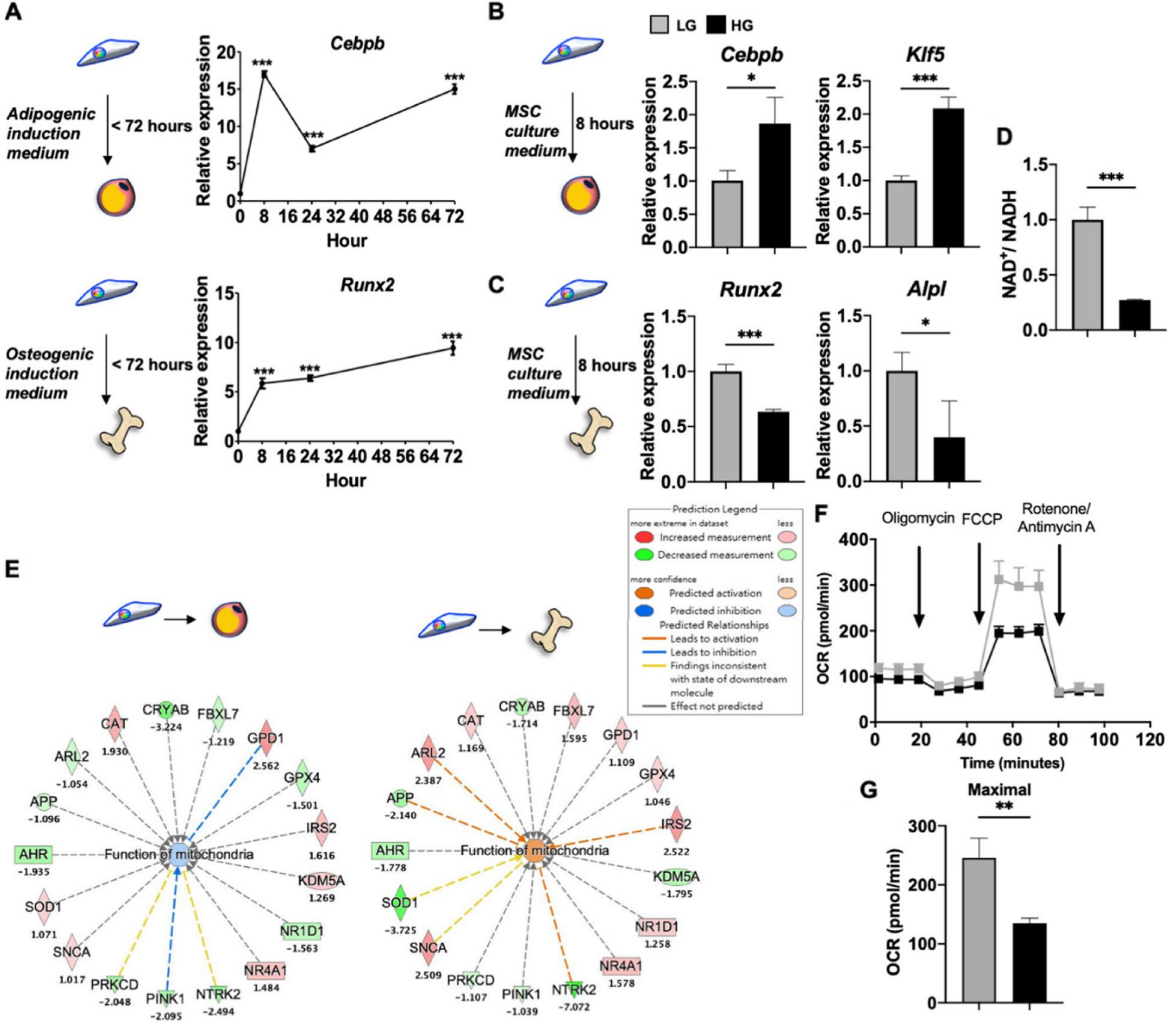


In the Funding section, we neglected to update the year-based numbering of two grants of B. L. Yen which we have updated.

This study … the Central Government S & T (NHRI‐13A1‐CSGP08‐048 to B.L.Yen), and the National Health Research Institutes (NHRI‐13A1‐ CSPP06‐014 to B.L.Yen).

The original publication has been corrected.
