# Supplementary material for: Excess glucose alone depress young mesenchymal stromal/stem cell osteogenesis and mitochondria activity within hours/days via NAD+/SIRT1 axis
Source: J Biomed Sci. 2024 May 13;31:49. doi: 10.1186/s12929-024-01039-0 (PMC11089752; doi:10.1186/s12929-024-01039-0)
Supplement: Supplementary file 1 — Supplementary Material 1. [file 12929_2024_1039_MOESM1_ESM.docx]

SUPPLEMENTARY INFORMATION

SUPPLEMENTARY METHODS

*Detection of intracellular reactive oxygen species (ROS) production*

Human bone marrow mesenchymal stem cells (BMMSCs) were incubated in culture medium (CM) containing low glucose (LG; 5.5 mM) or high glucose (25 mM) for 8, 24, or 48 hours, and then exposed to 10 μM of 2’-7’-dichlorofluoresceindiacetate (DCFDA, Sigma-Aldrich, MO, USA) for 30 minutes, following by flow cytometric analysis.

*Phosphorylated c-Jun N-terminal kinase (p-JNK) and total JNK protein detection*

Human BMMSCs were cultured in specific conditions for 8 hours, with subsequent extraction of total cellular protein for assessment of p-JNK and total JNK (both antibodies from Cell Signaling, MA, USA) expression with β-actin (Santa Cruz, CA, USA) as an internal control by western blot analyses.

*In vivo mice experimentation: longer term & blood glucose measurement*

Animal experimentation was conducted following approved protocols by the Institutional Animal Care and Use Committee of the National Taiwan University (No. 20170426). 6-week-old C57BL/6 male mice were given drinking water with added glucose prepared by adding 13g D-glucose (Sigma) into 100 ml drinking water [1] for 3 weeks. Blood glucose was measured at hour 4, days 2 and 6 [2, 3]. After 3 weeks, mice were sacrificed with BM cells harvested from one femur, and 50% of cells were stained for anti-CD45, anti-Lin (CD3, B220, CD11b, Ly6G, TER-119), anti-CD31, anti-Sca-1, and anti-CD51 or anti-perilipin-1 and anti-CD36 (all antibodies from BioLegend, San Diego, CA, USA), with flow cytometric analysis. The other 50% of BM cells were utilized for quantitative assessment of oil droplet accumulation by Oil Red O staining or alkaline phosphatase activity by incubating the protein lysates with substrate p-nitrophenylphosphate (pNPP, Sigma-Aldrich) [4]. The other femur was fixed with 10% formalin overnight and decalcified with 10% EDTA for 10 days. Then, paraffin embedding was performed and 5 μm sections were prepared for histological analyses with hematoxylin and eosin (H&E). For discontinuation of glucose experiments, 6-week-old C57BL/6 male mice were given drinking water with added glucose prepared by adding 13g D-glucose (Sigma) into 100 ml drinking water for 1 day and then discontinued with reversion back to regular water for 1 day or 5 days. Mice were euthanized, with uncultured CD45- BM cells harvested for RNA extraction and assessment of gene expression as well as JC-1 staining for assessment of mitochondrial function.

REFERENCE

1. Cheng CW, Biton M, Haber AL, Gunduz N, Eng G, Gaynor LT, Tripathi S, Calibasi-Kocal G, Rickelt S, Butty VL, et al. Ketone Body Signaling Mediates Intestinal Stem Cell Homeostasis and Adaptation to Diet. Cell. 2019;178:1115-1131 e15.

2. Furman BL. Streptozotocin-Induced Diabetic Models in Mice and Rats. Curr Protoc Pharmacol. 2015;70:5 47 1-5 47 20.

3. Zhan X, Wang L, Wang Z, Chai S, Zhu X, Ren W, Chang X. High-glucose administration induces glucose intolerance in mice: a critical role of toll-like receptor 4. J Clin Biochem Nutr. 2019;64:194-200.

4. Lin CH, Li NT, Cheng HS, Yen ML. Oxidative stress induces imbalance of adipogenic/osteoblastic lineage commitment in mesenchymal stem cells through decreasing SIRT1 functions. J Cell Mol Med. 2018;22:786-796.

SUPPLEMENTARY FIGURES

SUPPLEMENTAL FIGURE 1


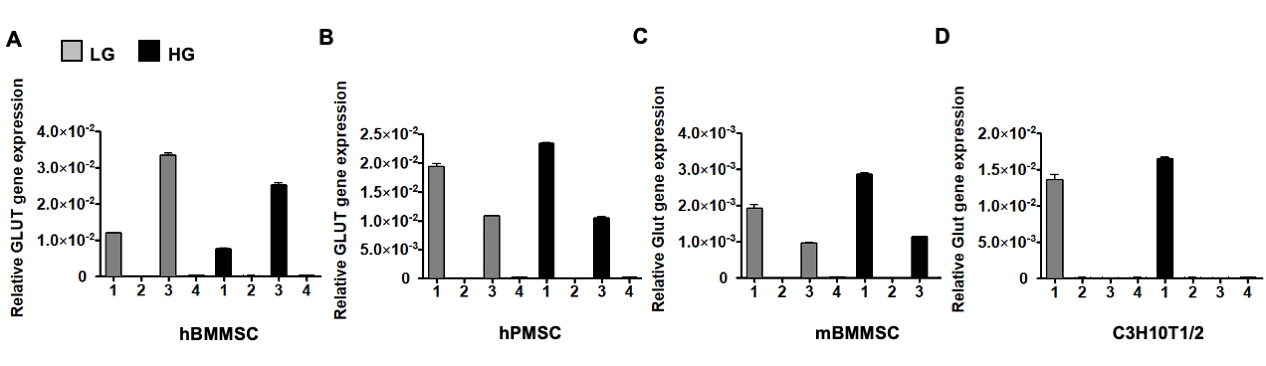


**Figure S1. Both human and murine mesenchymal stem cells (MSCs) predominantly express insulin-independent glucose transporters. Related to Figure 2.** qPCR analyses of glucose transporter (*GLUT*) 1 to 4 in primary human bone marrow MSCs (A, BMMSCs), primary human placental MSCs (B, PMSCs), primary mouse BMMSCs (C), and mouse C3H10T1/2 mesenchymal progenitor/stem cells (C3H; D), treated with low glucose (LG; 5.5 mM) or high glucose (HG; 25 mM) for 3 days. Gray bars represent data from LG-treated groups, while black bars represent data from HG-treated groups (n = 3 for each group in all figures).

SUPPLEMENTAL FIGURE 2


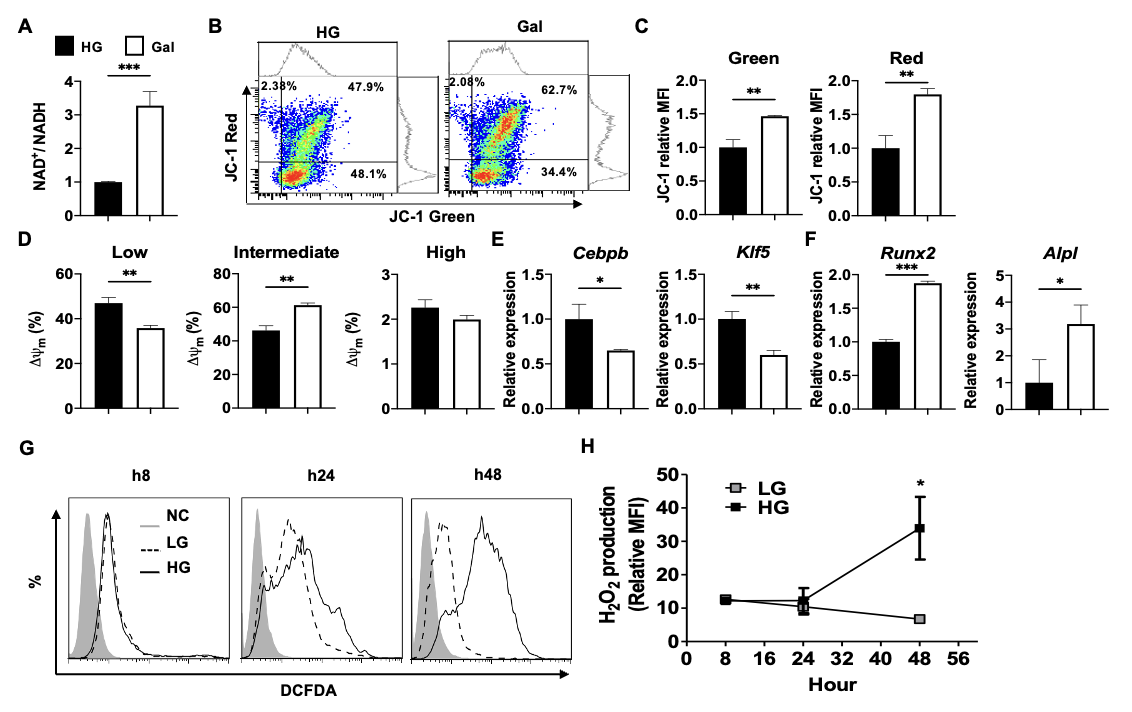


**Figure S2. Galactose enhances nicotinamide adenine dinucleotide*(*NAD*^+^*)-mediated mitochondrial metabolism with a shift of MSC commitment from adipogenesis to osteogenesis. Related to Figure 4.** (A) Ratio of cellular NAD^+^ to NADH (NAD^+^/NADH) as assessed in C3H with CM containing HG or galactose (Gal; 10 mM) for 8 hours. Cellular NAD^+^/NADH was normalized to the ratio in HG-cultured cells (n = 4 for each group). (B) Representative data for frequency analyses of low, intermediate and high mitochondrial activity as assessed in C3H with CM containing HG or Gal by flow cytometry at hour 8. C3H cells were cultured in CM with HG or Gal for 8 hours, and then treated with 2.5 μM of JC-1 for another 30 minutes. Gating for fluorescent signals of green^+^/red^-^, green^+^/red^+^ and green^-^/red^+^ was performed for frequency analyses of low, intermediate and high mitochondrial membrane potential (ΔΨM) in C3H, respectively. (C) Pooled data of depolarized and polarized mitochondrial ΔΨM as assessed in C3H with CM containing HG or Gal by analyzing green intensity and red intensity, respectively. Intensity of each signal was normalized to that in HG-treated C3H. (D) Pooled data for frequency analyses of low, intermediate and high mitochondrial activity in C3H with CM containing HG or Gal. (E and F) qPCR analyses of early adipogenic genes (E), CCAAT/enhancer-binding protein beta (*Cebpb*) and Kruppel-like factor 5 (*Klf5*), as well as early osteogenic genes (F), Runt-related transcription factor 2 (*Runx2*) and alkaline phosphatase (*Alpl*), as assessed in C3H treated with HG or Gal for 8 hours. Expression of each gene was normalized to the levels in HG-cultured C3H. Black bars represent data from HG-treated groups, while white bar represents data from Gal-treated groups (n = 3 for each group in all figures except figure A). (G) Representative and (H) pooled data for reactive oxygen species (ROS) production in LG- or HG-cultured human BMMSCs at 8, 24 and 48 hours using 2',7'-dichlorofluorescin diacetate (DCFDA) staining and flow cytometric analyses. Data are shown as mean ± SD. *, *p* < 0.05; **, *p* < 0.01; ***, *p* < 0.001.

SUPPLEMENTAL FIGURE 3


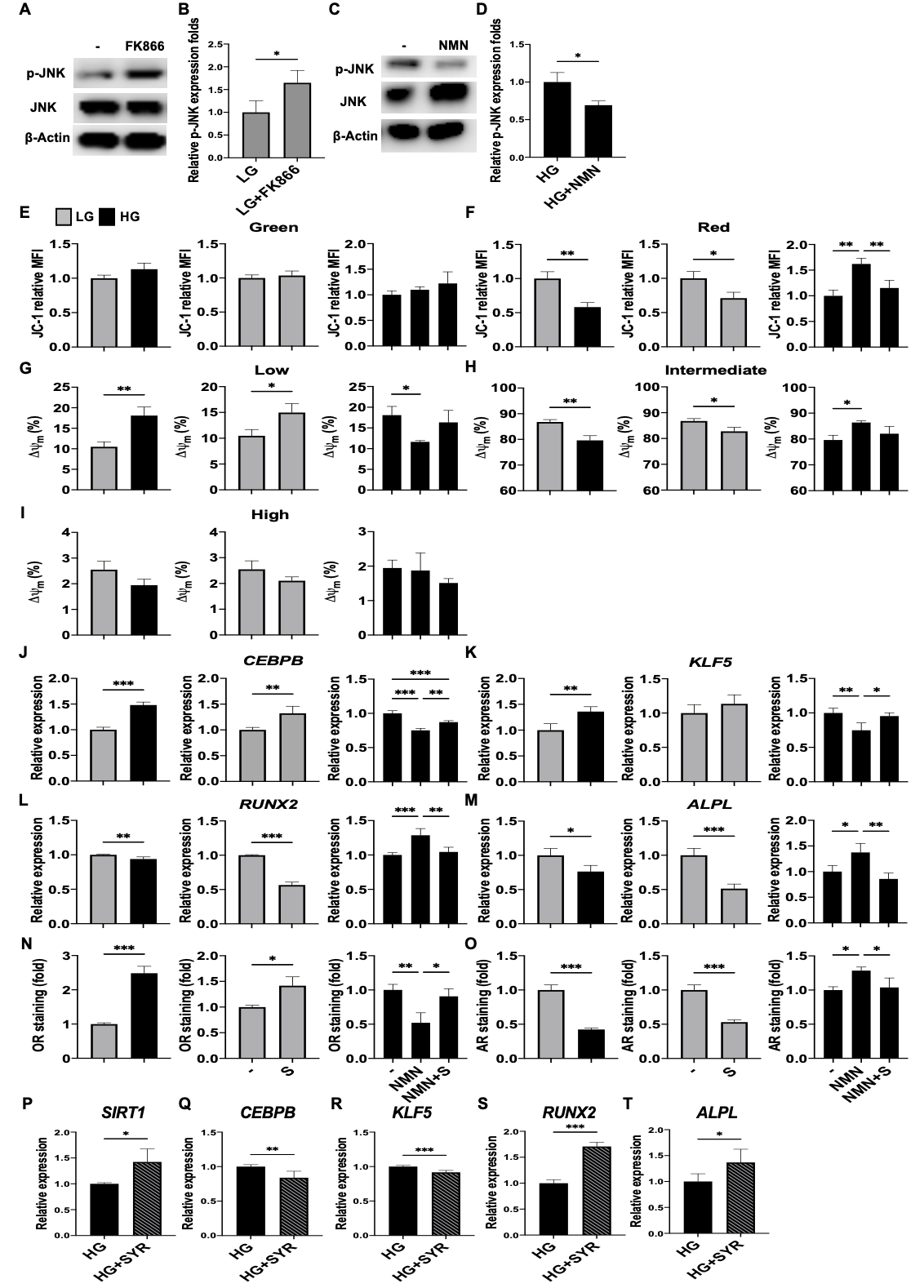


**Figure S3. HG suppresses mitochondrial activity and MSC osteogenesis through NAD^+^-dependent sirtuin (SIRT) 1. Related to Figure 5.** (A) Representative and (B) pooled data of phosphorylated c-Jun N-terminal kinase (p-JNK) and total JNK as assessed by western blot of human BMMSCs cultured in LG-CM in the presence or absence of FK866. Pooled protein levels were normalized to β-actin (n = 3 for each group). (C) Representative and (D) pooled data of p-JNK and total JNK as assessed by western blot of in human BMMSCs cultured in HG-CM in the presence or absence of NMN. Pooled protein levels were normalized to β-actin (n = 3 for each group). (E and F) Pooled data of depolarized (E) and polarized (F) mitochondrial ΔΨM as assessed in human BMMSCs with culture medium containing LG or HG (left), LG with or without sirtinol (S; 20 μM, middle), or HG with or without nicotinamide mononucleotide (NMN; 50 μM) or NMN combined with S (right) at hour 8. Intensity of each signal was normalized to that in human BMMSCs with LG alone (left and middle) or HG alone (right) (n = 3 for each group). (G-I) Pooled data for frequency analyses of low (G), intermediate (H) and high (I) mitochondrial activity as assessed in human BMMSCs with CM containing LG or HG (left), LG with or without S (middle), or HG with or without NMN or NMN combined with S (right) at hour 8 (n = 3 for each group). (J-M) qPCR analyses of *CEBPB* (J), *KLF5* (K), *RUNX2* (L), and *ALPL* (M) as assessed in human BMMSCs with CM containing LG or HG (left), LG with or without S (middle), or HG with or without NMN or NMN combined with S (right) at hour 8. Expression of each gene was normalized to the level in human BMMSCs with LG alone (left and middle) or HG alone (right) (n = 4 for each group). (N) Pooled data of assessment of oil drop accumulation with Oil Red (OR) staining in human BMMSCs treated with adipogenic medium containing LG or HG (left), LG with or without S (middle), or HG with or without NMN or NMN combined with S (right) for 3 days. The amount of OR staining was normalized to that in human BMMSCs with LG alone (left and middle) or HG alone (right) (n = 3 for each group). (O) Pooled data of assessment of extracellular mineralization with Alizarin Red (AR) staining in human BMMSCs treated with osteogenic medium containing LG or HG (left), LG with or without S (middle), or HG with or without NMN or NMN combined with S (right) for 28 days. The amount of AR staining was normalized to that in human BMMSCs with LG alone (left and middle) or HG alone (right) (n = 3 for each group). Gray bars represent data from LG-treated groups, while black bars represent data from HG-treated groups. (P) qPCR analysis of SIRT1 as assessed in human PMSCs cultured in HG-CM in the presence or absence of syringaresinol (SYR) at hour 8. Expression levels were normalized to the level in human PMSCs without SYR (n = 3 for each group). (J-M) qPCR analysis of *CEBPB* (J), *KLF5* (K), *RUNX2* (L), and *ALPL* (M) as assessed in human PMSCs cultured in HG-CM in the presence or absence of SYR at hour 8. Expression of each gene was normalized to the level in human PMSCs cultured in HG-CM alone (n = 6 for each group). Data are shown as mean ± SD. *, *p* < 0.05; **, *p* < 0.01; ***, *p* < 0.001.

SUPPLEMENTAL FIGURE 4


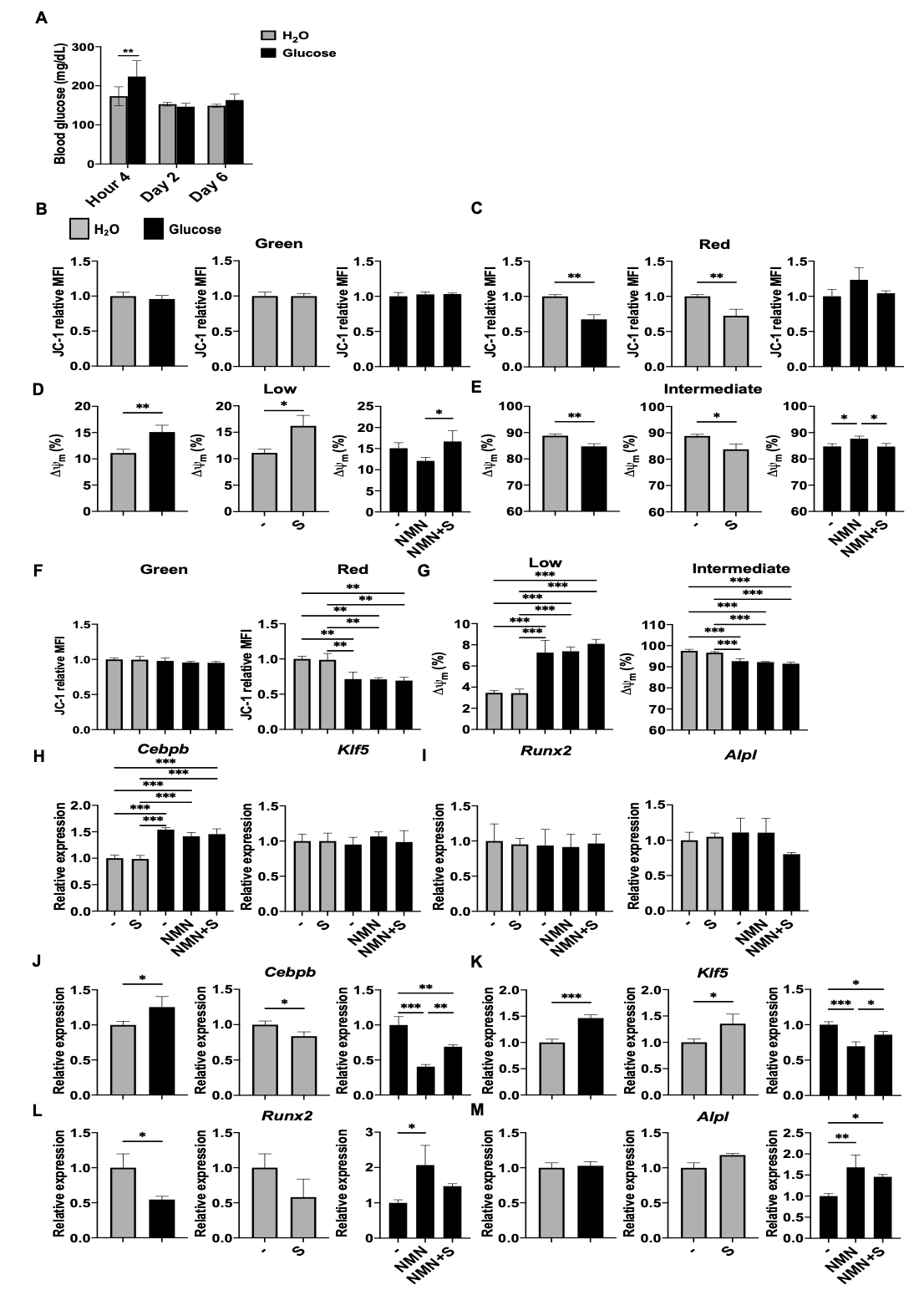


**Figure S4. *In vivo* HG intake rapidly downregulates mitochondrial activity and favors adipogenesis over osteogenesis in CD45^-^ BM cells. Related to Figure 6.** (A) Blood glucose levels as measured in 6-week-old mice given drinking water containing glucose or not at hour 4, days 2, and 6 (n = 6 for each time point). (B and C) Pooled data of depolarized (B) and polarized (C) mitochondrial ΔΨM as assessed in CD45^-^ BM cells of mice given by drinking water containing glucose or not (left), normal drinking water combined with intraperitoneal (i.p.) administration of S or not (middle), or glucose-contained drinking water with or without NMN or NMN combined with i.p. injection of S (right). 6-week-old mice were treated with or without D-glucose (13 g/ 100 ml H_2_O) or/and NMN (0.2 g/ 100 ml H_2_O) in drinking water combined with or without i.p. injection of S (300 mg/Kg/day) for 1 day, and then BM cells were harvested for staining with JC-1 and anti-CD45 for 30 minutes, with CD45^-^ cells gated for JC-1 analyses by flow cytometry. Fluorescent intensity of green and red was analyzed for assessment of basal and active ΔΨM, respectively, in CD45^-^ BM cells. Intensity of each signal was normalized to that in CD45^-^ BM cells isolated from mice with normal drinking water alone (left and middle) or glucose-contained drinking water alone (right). (D and E) Pooled data for frequency analyses of low (green^+^/red^-^) and intermediate mitochondrial (green^+^/red^+^) activity as assessed in CD45^-^ BM cells isolated from mice treated with drinking water containing glucose or not (left), normal drinking water combined with i.p. administration of S or not (middle), or glucose-contained drinking water with or without NMN or NMN combined with i.p. injection of S (right) for 1 day. (F and G) Pooled data of low and high mitochondrial ΔΨM (F) as well as frequency analyses of low and intermediate mitochondrial activity (G) as assessed in CD45^-^ BM cells isolated from mice treated with or without D-glucose or/and NMN in drinking water combined with or without i.p. injection of S for 5 days. Intensity of each signal in figure (F) was normalized to that in CD45^-^ BM cells isolated from mice with normal drinking water. (H and I) qPCR analyses of *Cebpb* and *Klf5* (H) as well as *Runx2* and *Alpl* (I) in BM cells isolated from mice treated with or without D-glucose or/and NMN in drinking water combined with or without i.p. injection of S for 1 day. Expression of each gene was normalized to that in BM cells isolated from mice with normal drinking water only. (J-M) qPCR analyses of *Cebpb* (J), *Klf5* (K), *Runx2* (L), and *Alpl* (M) in BM cells isolated from mice treated with drinking water containing glucose or not (left), normal drinking water combined with i.p. injection of S or not (middle), or glucose-contained drinking water with or without NMN or NMN combined with i.p. injection of S (right) for 5 days. Expression of each gene was normalized to that in BM cells isolated from mice with normal drinking water alone (left and middle) or glucose-contained drinking water alone (right). Gray bars represent data from mice treated with normal drinking water, while black bars represent data from mice treated with glucose-contained drinking water (n = 3 for each group). Data are shown as mean ± SD. *, *p* < 0.05; **, *p* < 0.01; ***, *p* < 0.001.

SUPPLEMENTAL FIGURE 5


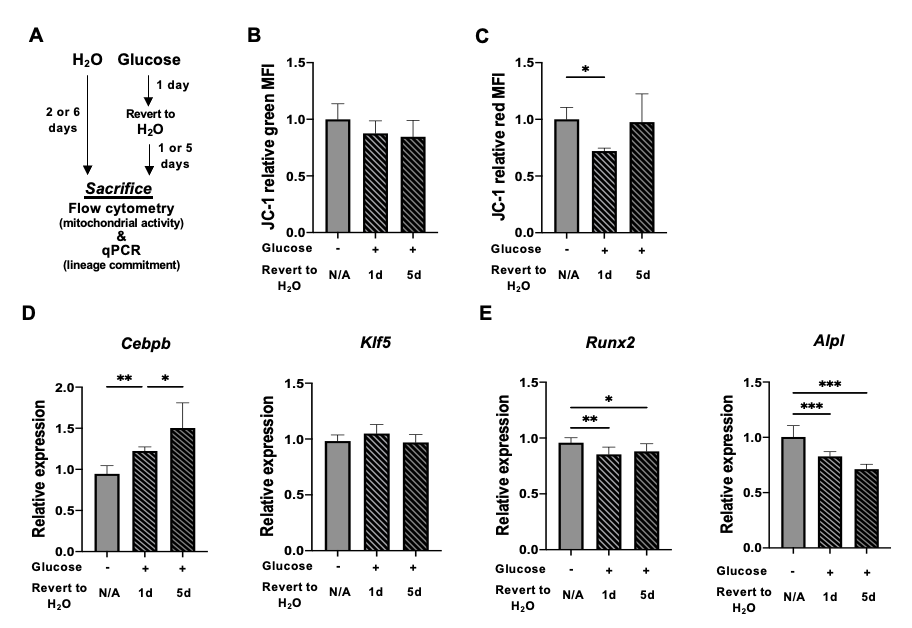


**Figure S5. MSC mitochondrial ΔΨM can be restored but not lineage commitment after 1 day of HG intake *in vivo*. Related to Figure 6.** (A) Experimental schema: BM cells were harvested from wildtype young adult C57BL/6 mice fed normal drinking water (2 groups, n = 6 in each group), or 1 day of drinking water with D-glucose (13 g/100 ml H_2_O) with reversion to normal drinking water for an additional 1 day (n = 6) or 5 days (n = 6), then (B & C) flow cytometric analyses of depolarized and polarized mitochondrial ΔΨM were performed on CD45^-^ cells (n = 6 for control group; n = 3 for glucose groups); and (D & E) qPCR analyses of *Cebpb* and *Klf5* (C) as well as *Runx2* and *Alpl* (D). Data are shown as mean ± SD. *, *p* < 0.05; **, *p* < 0.01; ***, *p* < 0.001.

SUPPLEMENTAL FIGURE 6


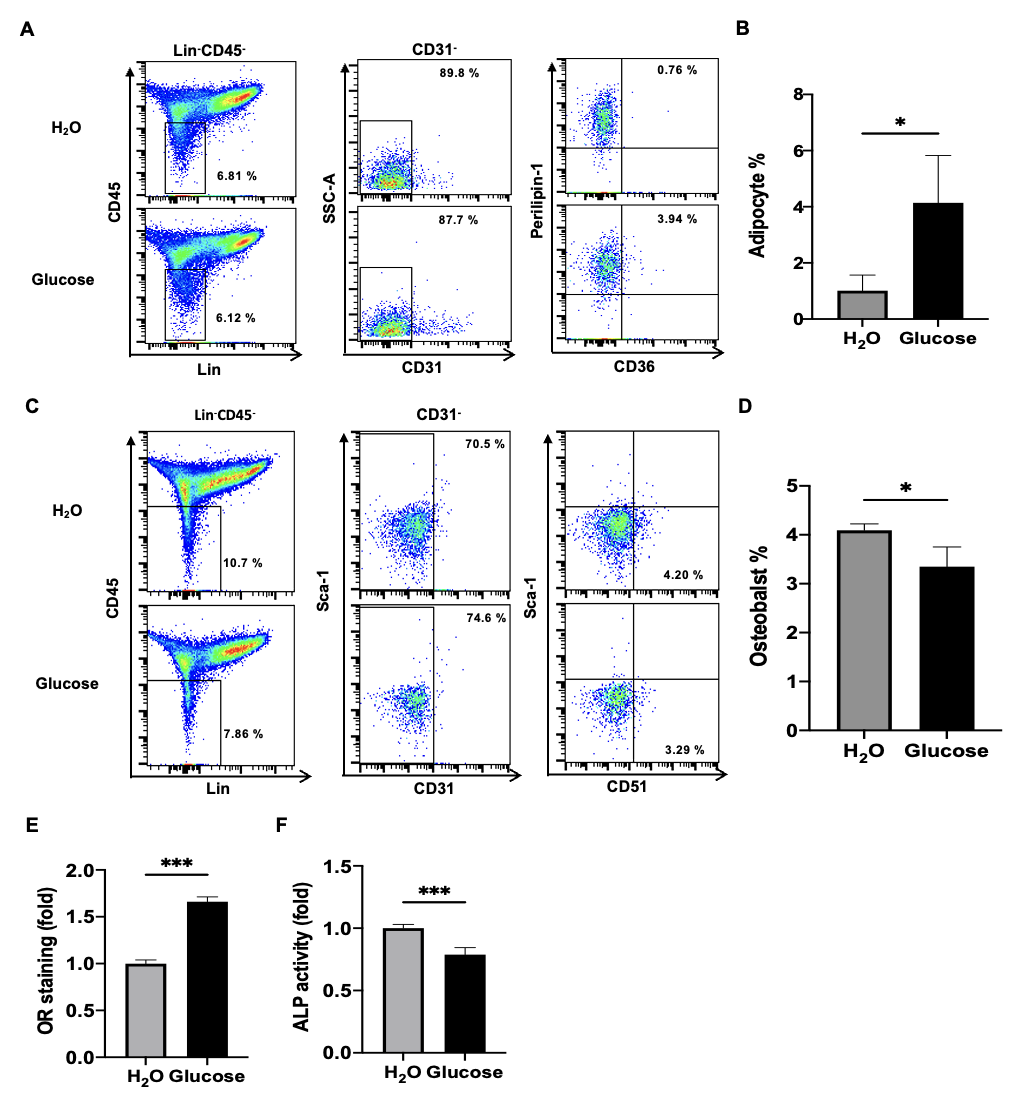


**Figure S**6**. HG intake *in vivo* after 3 weeks’ time decreases marrow osteoblasts but increases adipocytes. Related to Figure 6.** (A) Representative and pooled data for frequency analysis of adipocytes in BM as assessed with flow cytometry. 6-week-old mice were given drinking water with or without D-glucose (13 g/ 100 ml H_2_O) for 3 weeks, then sacrificed to harvest BM cells for flow cytometric analyses, with staining of anti-CD45, anti-Lin (CD3, B220, CD11b, Ly6G, TER-119), anti-CD31, anti-Sca-1, anti-perilipin-1, and anti-CD36. Single cells were gated for further analysis of adipocytes characterized by Lin^-^CD45^-^CD31^-^Sca-1^-^perilipin-1^+^CD36^+^ (n =3 for each group). (C and D) Representative and pooled data for frequency analysis of osteoblasts in harvested BM cells as assessed with flow cytometry, staining for anti-CD45, anti-Lin (CD3, B220, CD11b, Ly6G, TER-119), anti-CD31, anti-Sca-1,

anti-CD51. Single cells were gated for further analysis of osteoblasts characterized by Lin^-^CD45^-^CD31^-^Sca-1^-^CD51^+^ (n =3 for each group). (E and F) Oil droplet accumulation (E) and alkaline phosphatase (ALP) activity (F) as assessed in harvested BM cells by staining with Oil Red (OR) and enzymatic reaction, respectively (n = 3 for each group). Data are shown as mean ± SD. *, *p* < 0.05; ***, *p* < 0.001.
